# Dual effect of vitamin D_3_ on breast cancer-associated fibroblasts

**DOI:** 10.1186/s12885-024-11961-z

**Published:** 2024-02-15

**Authors:** Natalia Łabędź, Artur Anisiewicz, Martyna Stachowicz-Suhs, Joanna Banach, Dagmara Kłopotowska, Adam Maciejczyk, Patrycja Gazińska, Aleksandra Piotrowska, Piotr Dzięgiel, Rafał Matkowski, Joanna Wietrzyk

**Affiliations:** 1 Department of Experimental Oncology, Hirszfeld Institute of Immunology and Experimental Therapy, Weigla 12, 53-114 Wroclaw, Poland; 2 Łukasiewicz Research Network—PORT Polish Center for Technology Development, Stabłowicka 147, 54-066 Wrocław, Poland; 3 Department of Oncology, Wroclaw Medical University, Pl. Ludwika Hirszfelda 12, 53-413 Wroclaw, Poland; 4 Lower Silesian Oncology, Pulmonology and Hematology Center, Pl. Ludwika Hirszfelda 12, 53-413 Wroclaw, Poland; 5 Research Oncology, Division of Cancer Studies, Great Maze Pond, King’s College London, London, SE1 3SS UK; 6 Division of Histology and Embryology, Department of Human Morphology and Embryology, Wroclaw Medical University, Ul., Chałubińskiego 6a, 50-368 Wroclaw, Poland

**Keywords:** Tumor microenvironment, Breast cancer, Fibroblasts, Vitamin D_3_, Calcitriol

## Abstract

**Background:**

Cancer-associated fibroblasts (CAFs) play an important role in the tumor microenvironment. Despite the well-known in vitro antitumoral effect of vitamin D_3_ (VD_3_), its impact on breast CAFs is almost unknown. In this study, we analyzed the ex vivo effects of calcitriol on CAFs isolated from breast cancer tissues.

**Methods:**

CAFs were cultured with 1 and 10 nM calcitriol and their phenotype; gene expression, protein expression, and secretion were assessed. Calcitriol-treated CAFs-conditioned media (CM) were used to analyze the effect of CAFs on the migration and protein expression of MCF-7 and MDA-MB-231 cells.

**Results:**

Tumor tissues from VD_3_-deficient patients exhibited lower levels of β-catenin and TGFβ1, along with higher levels of CYP24A1 compared to VD_3_-normal patients. In VD_3_-deficient patients, CAF infiltration was inversely associated with CYP24A1 levels and positively correlated with OPN levels. Calcitriol diminished CAFs’ viability, but this effect was weaker in premenopausal and VD_3_-normal patients. Calcitriol reduced mRNA expression of *CCL2, MMP9, TNC*, and increased *PDPN, SPP1, and TIMP1*. It also decreased the secretion of CCL2, TNC, and the activity of MMP-2, while increasing cellular levels of TIMP1 in CAFs from all patient groups. In nonmetastatic and postmenopausal patients, PDPN surface expression increased, and CAFs CM from these groups decreased MCF-7 cell migration after ex vivo calcitriol treatment. In premenopausal and VD_3_-deficient patients, calcitriol reduced IDO1 expression in CAFs. Calcitriol-treated CAFs CM from these patients decreased OPN expression in MCF-7 and/or MDA-MB-231 cells. However, in premenopausal patients, calcitriol-treated CAFs CM also decreased E-cadherin expression in both cell lines.

**Conclusion:**

The effects of calcitriol on breast CAFs, both at the gene and protein levels, are complex, reflecting the immunosuppressive or procancer properties of CAFs. The anticancer polarization of CAFs following ex vivo calcitriol treatment may result from decreased *CCL2, TNC* (gene and protein), *MMP9*, and MMP-2, while the opposite effect may result from increased *PDPN, TIMP1* (gene and protein), and *SPP1*. Despite these multifaceted effects of calcitriol on molecule expression, CAFs’ CMs from nonmetastatic and postmenopausal patients treated ex vivo with calcitriol decreased the migration of MCF-7 cells.

**Supplementary Information:**

The online version contains supplementary material available at 10.1186/s12885-024-11961-z.

## Background

Crosstalk between cancer cells and components of the tumor microenvironment (TME) plays an essential role in breast cancer development and progression. The TME is composed of stromal, endothelial, and immune cells, as well as extracellular matrix (ECM) and soluble factors secreted by these cells [1]. Among all the components of the breast TME, cancer-associated fibroblasts (CAFs) constitute the most abundant cell type. By producing various factors, such as growth factors, cytokines, and proteases, CAFs control numerous processes that contribute to tumor initiation, growth, progression, and metastasis [1]. On the other hand, vitamin D_3_, more specifically its biologically active form, calcitriol, is known for its antiproliferative, antimetastatic, proapoptotic, prodifferentiation, and immunomodulatory effects on breast cancer in vitro [2].

In addition, a low plasma 25(OH)D_3_ concentration has been associated with breast cancer risk in both premenopausal [3] and postmenopausal women [4]. However, in older women (> 50 years old), a higher plasma 25(OH)D_3_ concentration was reported to be associated with a higher risk of breast cancer development [5]. In addition, Kanstrup et al. observed that plasma levels below 52 nmol/l (approximately 21 ng/ml) and above 99 nmol/l (40 ng/ml) led to inferior event-free survival [6]. Most breast cancer patients have lower plasma 25(OH)D_3_ concentrations than healthy women of the same age [7], and patients with advanced breast cancer or a more aggressive tumor phenotype (basal-like, ER-negative, or triple-negative) often exhibit even lower plasma 25(OH)D_3_ concentrations than patients in opposing groups (benign cancer, luminal-like, ER-positive) [8, 9]. According to other studies, high vitamin D receptor (VDR) expression in tumor tissue and low expression of CYP24A1, responsible for calcitriol degradation, are inversely associated with aggressive tumor characteristics, including large tumor size, ER- and PR-negativity, triple-negative subtypes, or high Ki67 expression [10–12]. However, Lopes et al. reported that VDR expression decreases with breast cancer development, and the sensitivity of cancer cells to calcitriol activity also decreases [12]. Moreover, clinical studies, like VITAL study (VITamin D and OmegA-3 TriaL) have not reported a relationship between vitamin D_3_ supplementation and breast cancer risk or incidence [2, 13].

CAFs are characterized by their spindle shape and the absence of epithelial, endothelial, or immune-cellular phenotypes [14]. Unlike normal fibroblasts, which are known for their role in wound healing or fibrosis, CAFs, once activated, remain permanently activated, aligning with the hypothesis of Dvorak et al. that “tumors are wounds that do not heal” [15]. CAFs play versatile roles in tumor progression. Through ECM remodeling, promoting neovascularity, inducing stem cell phenotype, cancer cell proliferation, migration, and invasion, CAFs take part in tumor resistance to therapy and support the development of metastasis [16]. Additionally, CAFs modulate cancer metabolism, tumor angiogenesis, and anticancer immunity through interactions with surrounding cells [17, 18].

Despite numerous research reporting the influence of vitamin D_3_ (and calcitriol or its analogs) on the cancer epithelial compartment, its impact on CAFs has been described only in several articles. To date, publications have described the effects of calcitriol or its analogs on CAFs in prostate cancer [19], pancreatic cancer [20–23], gastric cancer [24], colon cancer [25, 26], and breast cancer [27, 28]. The majority of these studies show that calcitriol or its analogs (1) reduces fibroblast activation (observed as a reduction in alpha-smooth muscle actin (αSMA) expression, collagen gel contractility, or the restoration of the resting state in stellate cells) [20, 22, 25, 26], (2) reduces the proliferation and migration of CAFs [20, 23, 26], (3) suppresses the promigratory and proinvasive abilities of CAFs on cancer cells [21, 25], (4) inhibits chemotherapy resistance in cancer cells induced by CAFs [20, 22, 24], (5) reduces the immunosuppressive activity of CAFs (T-cell activation and effector function) [23, 28], or (6) modulates the expression of genes and proteins related to adhesion, apoptosis, proliferation, migration, ECM remodeling, inflammatory response, or angiogenesis [19, 20, 25, 26, 28]. Furthermore, some VDR agonists (paricalcitol due to its normalization effect on stellate cells) are presently under investigation as a potential candidate for clinically targeting cancer-associated fibroblasts (CAFs) [14, 29]. However, our previous study indicated conflicting effects of vitamin D_3_ and calcitriol on the activation and activity of normal fibroblasts or CAFs in vivo. In 4T1-bearing mice, calcitriol administration resulted in lung fibroblasts that were more susceptible to activation by metastatic cancer cells than fibroblasts from mice on a vitamin D_3_-supplemented diet without calcitriol treatment. Moreover, in E0771-bearing mice, a vitamin D_3_-supplemented diet along with calcitriol treatment resulted in the development of CAFs with a similar phenotype, reflecting their increased procancerous activity [27]. However, in mice, breast cancer developed only for shorter periods (23–28 days) compared to humans (years). Long periods of TME development in humans could lead to the formation of CAFs that may respond differently to vitamin D_3_ [27].

Therefore, based on data described in previous paragraphs regarding the different relationships between vitamin D_3_ (25(OH)D_3_ plasma levels, VDR expression in cancer tissues, etc.) and the occurrence of breast cancer, as well as the CAFs importance in tumor development, we assumed that vitamin D_3_ or its active metabolite may have various impact on CAFs depending on their origin. This study aimed to investigate how calcitriol affects the cancer-promoting properties of CAFs isolated from breast cancer tissues of patients with different clinical characteristics such as pre or postmenopausal, vitamin D_3_-normal or vitamin D_3_-deficient, and patients without metastases or with metastatic tumors. Established primary cultures of CAFs isolated from patients were stimulated ex vivo with 1 nM or 10 nM calcitriol. The effects of calcitriol on the cell viability, expression, secretion, and activity of selected molecules in CAFs and their impact on cancer cells were evaluated.

## Materials and methods

### Patient population

Breast cancer tissue samples were collected from patients who underwent surgery at the Breast Cancer Unit of the Lower Silesian Oncology, Pulmonology, and Hematology Center in Wroclaw, Poland, between February 2019 and December 2020. Only patients who had not been treated with neoadjuvant therapy were included in the study. A total of 127 patients agreed to take part in this study, with 90% of tumors being estrogen-positive (ER^+^), 20% overexpressing human epidermal growth factor receptor 2 (HER2^+^), and 5% categorized as triple-negative breast cancers (TNBC). Furthermore, among the patients, tumors were categorized as follows: 16% were Grade 1, 64% were Grade 2, and 20% were Grade 3. Detailed patient information is available in Table S1 in the Supplementary Materials.

### Tissue sample preparation

Fresh surgical specimens were used to isolate CAFs and determine the expression of osteopontin (OPN), transforming growth factor β 1 (TGFβ1), β-catenin, VDR, CYP24A1 (cytochrome P450 family 24 subfamily A member 1), and CYP27B1 (cytochrome P450 family 27 subfamily B member 1) in tumor tissue. Clinical data, including age, plasma follicle-stimulating hormone (FSH) levels, plasma 25(OH)D_3_ levels, and the presence of lymph nodes or distant metastases, were collected and received from Lower Silesian Oncology, Pulmonology, and Hematology Center along with the tumor tissue samples. Plasma 25(OH)D_3_ levels and FSH levels were examined in a certified diagnostic center (Diagnostyka Sp. z o.o.). The tests were conducted using the Liaison analyzer, employing chemiluminescence technology for measuring 25(OH)D3 levels or the assay from Roche, which implements electroluminescence technology for measuring FSH levels. All participants provided written informed consent. All experimental protocols were approved by the Bioethical Committee at the Medical University of Wrocław, Poland (approval numbers: 603/2018 and 855/2019).

Patients with plasma 25(OH)D_3_ levels below 30 ng/mL were classified into the “deficiency” group, while those with levels above 30 ng/mL were categorized as “normal.” Patients with plasma FSH levels exceeding 25.8 mIU/mL were considered “postmenopausal,” while those below this threshold were considered “premenopausal.” Patients with metastases in regional lymph nodes or distant organs were assigned to the “metastatic” group, while patients without any metastases were assigned to the “nonmetastatic” group.

### Cell lines

Breast cancer MCF-7 cells were obtained from the European Collection of Authenticated Cell Cultures (ECACC, Salisbury, UK), and MDA-MB-231 cells were obtained from the American Type Culture Collection (ATCC, Rockville, MD, USA). MCF-7 cells represent ER^+^ breast cancers, characterized by ER and progesterone receptor (PR) positivity and the absence of HER2 expression. In contrast, MDA-MB-231 is a TNBC model, characterized by the absence of ER, PR, and HER2 expression. MCF-7 cells were cultured in Eagle’s Minimum Essential Medium (EMEM, Hirszfeld Institute of Immunology and Experimental Therapy, Polish Academy of Sciences (HIIET PAS), Wroclaw, Poland) supplemented with 10% (*v/v*) fetal bovine serum (FBS), 2.0 mM L-glutamine, 1% (*v/v*) nonessential amino acids, 8 μg/ml insulin, 100 μg/mL streptomycin (all from Sigma‒Aldrich, Saint-Louis, MO, USA), and 100 U/mL penicillin (Polfa Tarchomin S.A., Warsaw, Poland). MDA-MB-231 cells were cultured in RPMI + HEPES (HIIET PAS, Wroclaw, Poland) supplemented with 10% (*v/v*) FBS, 2.0 mM L-glutamine, 100 μg/mL streptomycin (all from Sigma‒Aldrich, Saint-Louis, MO, USA), and 100 U/mL penicillin (Polfa Tarchomin S.A., Warsaw, Poland). The cells were incubated at 37 °C in a humid atmosphere with 5% CO_2_.

### Western blot analysis of selected proteins in tumor tissue

Breast tumor specimens were frozen in liquid nitrogen and stored at − 80 °C. Samples were prepared from frozen tissue and subsequently transferred to tubes containing a homogenizing ball (Mp Biomedicals LLC., Santa Ana, CA, USA) and radioimmunoprecipitation assay (RIPA) buffer with a cocktail of phosphatase and protease inhibitors (both from Sigma‒Aldrich, Saint-Louis, MO, USA). Homogenization was carried out using a Fast Prep®-24 MP Bio homogenizer (MP Biomedicals, Santa Ana, CA, USA). After homogenization, the samples were centrifuged at 10 000 × g for 10 min at 4 °C, and supernatants were transferred to fresh Eppendorf tubes. The protein concentration in the homogenates was measured using the Quick Start™ Bio-Rad Protein Assay (Bio-Rad, Hercules, CA, USA).

Polyacrylamide gel electrophoresis was carried out on 50 μg of protein samples. The proteins were transferred onto polyvinylidene difluoride (PVDF) membranes with a pore size of 0.45 μm (Merck Millipore, Billerica, MA, USA). After incubating for 1 h with 5% nonfat dry milk in 0.1% Tris-buffered saline/Tween-20 (HIIET PAS, Wroclaw, Poland/Sigma‒Aldrich, Saint-Louis, MO, USA), the membranes were incubated overnight at 4 °C with the following antibodies (at appropriate dilutions: rabbit anti-OPN polyclonal antibody (1:1000, Proteintech, Rosemont, IL, USA), rabbit anti-TGFβ1 antibody (1:500, Proteintech, Rosemont, IL, USA), rabbit anti-β-catenin antibody (1:2000, Santa Cruz Biotechnology Inc., Dallas, TX, USA), rabbit anti-VDR antibody (1:1000, Bioss Antibodies, Woburn, MA, USA), rabbit anti-CYP24A1 antibody (1:500, Abcam, Cambridge, UK), and anti-CYP27B1 antibody (1:1000, Abcam, Cambridge, UK). The next day, the membranes were washed and incubated with secondary mouse antirabbit immunoglobulin G–horseradish peroxidase (HRP) monoclonal antibody (1:10 000, Santa Cruz Biotechnology Inc., Dallas, TX, USA) for 1 h. Chemiluminescence was induced using Clarity Western ECL Substrate (Bio-Rad, Hercules, CA, USA), and protein detection was performed with a ChemiDoc Imaging System (Bio-Rad, Hercules, CA, USA). Subsequently, the membranes were incubated with 100% methanol for 30 min at room temperature (RT; Avantor Performance Materials Poland, Gliwice, Poland). The membranes were then washed, blocked for 1 h, washed again, and incubated with mouse anti-β-actin-HRP (C4) monoclonal antibody (1:5000, Santa Cruz Biotechnology, Dallas, TX, USA) for 1 h at RT. Detection of proteins was performed as described above. Densitometry analysis was carried out in ImageJ software with the tested protein normalized to β-actin.

### Hematoxylin and eosin staining of tumor tissues and assessment of CAF activation

Formalin-fixed paraffin-embedded (FFPE) tissue blocks containing tumor tissue, prepared by the Lower Silesian Center for Oncology, Pulmonology, and Hematology, were further processed at the Department of Histology at Wroclaw Medical University. Deparaffinized and rehydrated slides were stained with Maye’s hematoxylin (cat. no. 05–06002/L, Bio-Optica, Milano, Italy) for 3 min, followed by rinsing in running water for 10 min. The next step was to stain slides with 1% aqueous eosin solution (cat. no. 05–10002/L, Bio-Optica) for 10 min. Immediately after staining, all slides were dehydrated in graded ethanol concentrations (70%, 96%, absolute) and mounted with Euparal (cat. no. 7356.1, Karlsruhe, Germany). Digital images were captured using a Nanozoomer S60 scanner (Hamamatsu, Japan) at a magnification of × 40 (0.23 μm/pixel) for the semi-quantitative assessment of fibroblast infiltration into the tumor stroma. The assessment was conducted independently by two histopathology scientists, N.L. and P.G. CAFs were identified as large, plump spindle-shaped cells each with a prominent nucleus, distinguishing them from normal fibroblasts, which are thin, wavy, and small spindle cells [30]. To estimate CAF infiltration, a detailed stromal CAF assessment was performed. It included the assessment of activation and density, rated on a 5-point scale, where 1 denoted low activation or density of fibroblasts, 2 denoted medium–low, 3 denoted medium, 4 denoted medium–high, and 5 denoted high activation/density of fibroblasts. To facilitate further assessments, the level of density and the level of fibroblast activation were combined into a single score called the CAF infiltration score. A detailed description of the scoring algorithm is provided in the Supplementary Materials (Table S2).

### Establishment of primary cell cultures

Harvested tissues were cut into approximately 1 mm pieces and subjected to 14–22 h (overnight) of digestion at 37 °C with gentle shaking. The digestion solution consisted of complete medium (RPMI, 5% (*v/v*) FBS (both Sigma‒Aldrich, Saint-Louis, MO, USA), 5% (*v/v*) horse serum (HS, Gibco, Grand Island, NY, USA), 4.0 mM L-glutamine, 2.5 g/L glucose, 1 mM pyruvate, 1% (*v/v*) nonessential amino acids, 100 μg/mL streptomycin (all Sigma‒Aldrich, Saint-Louis, MO, USA) and 100 U/mL penicillin (Polfa Tarchomin S.A., Warsaw, Poland)), 1 mg/mL collagenase IV (collagenase from Clostridium histolyticum; Sigma‒Aldrich, Saint-Louis, MO, USA), and 1 mg/mL DNase I (Roche, Basel, Switzerland). Following digestion, homogenates were filtered through a 70 μm mesh strainer (EASYstrainer™, Greiner Bio-One, Kremsmünster, Austria) and then washed with fresh phosphate-buffered saline (PBS) containing 2% (*v*/*v*) FBS. The cell suspension was then centrifuged for 7 min (4 °C, 350 × *g*) followed by erythrocyte lysis. In brief, cell pellets were resuspended in 1 mL of lysis buffer (Sigma‒Aldrich, Saint-Louis, MO, USA) and shaken for 1 min; then, PBS with serum was added, and the suspension was centrifuged for 7 min (4 °C, 350 × *g*). The resulting cell pellets were resuspended in 5 mL of fresh PBS with FBS, and cell quantity was determined by counting in a Bürker chamber in a trypan blue solution (0.4% (*w*/*v*) Sigma‒Aldrich, Saint-Louis, MO, USA).

CAFs were isolated using Anti-Fibroblast MicroBeads (Miltenyi Biotec, Auburn, CA, USA) according to the manufacturer’s protocol. In brief, centrifuged pellets (7 min, 4 °C, 350 × g) were resuspended in separation buffer containing PBS at pH 7.2, 0.5% (*v/v*) bovine serum albumin, 2 mM ethylenediaminetetraacetic acid (Sigma‒Aldrich, Saint-Louis, MO, USA), and TruStain FcX (antimouse CD16/CD32) antibody (BioLegend, San Diego, CA, USA) and then incubated for 10 min at 4 °C to block Fc receptors (0.1 µg/100 µL volume). After blocking, magnetic beads were added (20 µL/10^6^ of total cells) to the cells and incubated for 30 min in the dark at RT. Subsequently, 1 mL of separation buffer was added to the cells, followed by centrifugation (7 min, 4 °C, 350 × g). The cell pellets were resuspended in 1 mL of separation buffer and applied onto activated MS columns (Miltenyi Biotec, Auburn, CA, USA) placed in the magnetic field of the MiniMACS Separator (Miltenyi Biotec, Auburn, CA, USA). After three washes with 500 µL of separation buffer, the columns were transferred into new sterile 10 mL tubes, and cells were flushed into collection tubes. The collected cells were counted using a Bürker chamber in trypan blue solution (0.4% (w/v)) and allocated for CAF phenotype assessment using flow cytometry or seeded onto 24-well plates in CAFs medium (Ham’s F12 (Corning, New York, NY, USA), 5% FBS (Sigma‒Aldrich, Saint-Louis, MO, USA), 5% HS (Gibco, Grand Island, NY, USA), 10 μg/mL insulin, 0.5 μg/mL hydrocortisonum, 0.05 μg/mL cholera toxin, 20 ng/mL EGFh, 100 μg/mL streptomycin (all Sigma‒Aldrich, Saint-Louis, MO, USA), 100 U/mL penicillin (Polfa Tarchomin S.A., Warsaw, Poland), basic fibroblast growth factor (bFGF, BioLegend, San Diego, CA, USA) and 10 μL/5 mL Primocin (InvivoGen, San Diego, CA, USA)). When the cells reached confluence, they were transferred into tissue flask cells (Sarstedt, Nümbrecht, Niemcy) for further testing.

### Flow cytometry

Freshly isolated CAFs were used to evaluate the purity of the culture and cell phenotype. Cell suspensions of 1.5–5 × 10^4^ cells per sample were resuspended in pure PBS and incubated with eBioscience™ Fixable Viability Dye eFluor™ 780 (Invitrogen, Waltham, MA, USA) for 30 min at 4 °C. Cell surface markers were stained extracellularly in FACS buffer (2% (*v*/*v*) FBS in PBS) for 30 min at 4 °C. To carry out intracellular staining, cells were fixed in a fixation buffer (BioLegend, San Diego, CA, USA) for 20 min at RT, washed, and permeabilized using Intracellular Staining Perm Wash Buffer (BioLegend, San Diego, CA, USA) three times (7 min, 20 °C, 350 × *g*). The samples resuspended in the FACS buffer were analyzed using a BD LSR Fortessa cytometer with FACSDiva V8.0.1 software (BD Biosciences, Franklin Lakes, NJ, USA). For each marker, the median fluorescence intensity (MFI) of stained cells relative to the isotype control was determined. The following antibodies were used for CAF staining: α-smooth muscle actin (α-SMA)-PE (Abcam, Cambridge, UK), CD31-BV-421, CD45-FITC, EpCAM-PE/Cy7, platelet-derived growth factor receptor β (PDGFRβ)-APC, fibroblast specific protein 1 (FSP1)-PerCP/Cy5.5, Podoplanin (PDPN)-PE/Dazzle™ 594 (all Biolegend, San Diego, CA, USA), and Tenascin C (TNC)-Alexa Fluor 700 (Novus Biologicals, Centennial, CO, USA). The same protocol was applied to established cultures after calcitriol treatment.

### Immunofluorescence staining

A total of 0.5–2.0 × 10^3^ cells/well were cultured for imaging on a Falcon® 96-well Black/Clear Flat Bottom TC-treated Imaging Microplate (Corning, New York, NY, USA) for 72 h. When cells reached the appropriate confluence, their surface was washed with a PBS solution, fixed in freshly prepared 4% (*v*/*v*) paraformaldehyde (Avantor Performance Materials Poland, Gliwice, Poland) for 10–15 min, washed with PBS, and permeabilized in 0.25% (*v*/*v*) Triton X-100 (Sigma‒Aldrich, Saint-Louis, MO, USA) for 15 min at RT. Then, the cells were washed and blocked for 30 min in 1% (*w*/*v*) bovine serum albumin (Sigma‒Aldrich, Saint-Louis, MO, USA) solution in 0.1% (*v*/*v*) PBS/Tween 20 (Sigma‒Aldrich, Saint-Louis, MO, USA) at RT. Next, the fixed cells were incubated with primary antibodies against vimentin (NBP1-31327, dilution 1:500; Novus Biologicals, Centennial, CO, USA) in a blocking solution at 4 °C overnight. Next, after washing with PBS, a secondary antibody (anti-rabbit antibody Alexa Fluor 488, ab150077; Abcam, Cambridge, UK) in the blocking solution was used for 1 h at RT. Finally, the samples were rinsed with PBS and photographed using an Olympus IX81 fluorescence microscope (Olympus, Warsaw, Poland) with CellSense software (Olympus, Warsaw, Poland).

### Cell viability assay

Twenty-four hours prior to the addition of calcitriol, CAFs were seeded in 96-well plates (2 × 10^4^ cells/well). Then, the cells were treated with four different concentrations of calcitriol, ranging from 1 to 10^3^ nM, or its solvent, EtOH. After an incubation period of 72 h, the cells were fixed for 1 h with cold 50% trichloroacetic acid, washed five times with tap water, and then stained with 0.4% sulforhodamine B (in 1% acetic acid) for 30 min. Unbound dye was removed by rinsing the plates four times with 1% acetic acid., The protein-bound dye was extracted with a 10 mM unbuffered Tris base, and the optical density (*λ* = 540 nm) was determined in a computer-interfaced BioTek Synergy H4 Hybrid Microplate Reader. Cell viability was calculated using the following formula:

$$\mathrm{cell}\;\mathrm{viability}\;\left(\%\right)=\frac{\text{Ap}-\text{Am}}{\text{Ac}-\text{Am}}\times100$$

where Ap is the absorbance of cells treated with compounds, Am is the absorbance of the control media, and Ac is the absorbance of the control cells.

### Ex vivo calcitriol treatment

A total of 3 × 10^5^ cells/plate were seeded on 10 cm culture plates in triplicate. After 24 h, the medium was replaced with a medium containing calcitriol (0, 1, or 10 ng/mL, Cayman Chemical, Ann Arbor, MI, USA). CAFs were stimulated with calcitriol for 72 h, and then cell lysates and cell supernatants were collected for further tests.

### Gene expression in CAF lysates

Total RNA was extracted using 1 mL of Tri-reagent (Sigma Aldrich, Saint-Louis, MO, USA), followed by RNA purification with Direct-zol™ RNA Miniprep (ZYMO RESEARCH, Tustin, CA, USA) according to the manufacturer’s protocol. RNA was then retrotranscribed using SuperScript™ IV VILO Master Mix (Invitrogen, Waltham, MA, USA).

To identify genes whose expression was altered by calcitriol treatment in CAFs, custom-made Human CAFs TaqMan™ Array Cards (Applied Biosystems, Waltham, Massachusetts, USA) were used. Using cDNA from 10 nM calcitriol-treated CAFs from tumors of 14 premenopausal and 5 postmenopausal patients, the expression of 62 genes (including 4 endogenous controls) was assessed (list of genes presented in Table S3 in Supplementary Materials). Reactions were prepared according to the manufacturer’s instructions (Applied Biosystems, Waltham, Massachusetts, USA) using 110 ng of cDNA for one port. The following program was used: 10 min at 95 °C for initial denaturation and 40 cycles at 95 °C for 15 s and 60 °C for 1 min, using a ViiA™ 7 Real-Time PCR System (Thermo Fisher Scientific, Waltham, MA, USA). Gene expression values were independently normalized vs two chosen housekeeping genes (GAPDH (Hs99999905_m1) and RPLP0 (Hs99999902_m1)) and the respective untreated control (cells not treated with calcitriol) using the comparative ΔΔCt method in QuantStudio™ Real-Time PCR Software and ExpressionSuite Software (Thermo Fisher Scientific, Waltham, MA, USA).

Following this, the expression of the following genes chosen in screening PCR was determined in CAFs treated with 1 nM and 10 nM calcitriol using ready-to-use primers and probes (TaqMan® Gene Expression Assays; Thermo Fisher Scientific, Waltham, MA, USA): C–C motif chemokine ligand 2 (*CCL2*, Hs00234140_m1), metalloproteinase 9 (*MMP9*, Hs00234579_m1), *PDPN* (Hs00366766_m1), secreted phosphoprotein (*SPP1*, Hs00959010_m1), tissue metalloproteinase inhibitor 1 (*TIMP1*, Hs00171558_m1), *TNC* (Hs01115664_m1), and *VDR* (Hs01045840_m1). Quantitative PCR (qPCR) was performed using 20 × presented probes, 50 ng cDNA, and 2 × TaqMan™ Gene Expression Master Mix (Thermo Fisher Scientific, Waltham, MA, USA) in a ViiA™ 7 Real-Time PCR System (Thermo Fisher Scientific, Waltham, MA, USA) as described above.

### Enzyme-linked immunosorbent Assays (ELISA) in CAF supernatants

After incubation with calcitriol, media from CAFs’ cultures were collected and centrifuged (400 × g, 10 min, 4 °C). ELISAs were performed according to the manufacturer’s protocols. The expression of the following proteins was measured in supernatants: C-X-C motif chemokine ligand 12 (CXCL12), hepatocyte growth factor (HGF) (both Biorbyt, Cambridge, UK), CCL2, MMP9, OPN and TNC (Invitrogen, Waltham, MA, USA). The results obtained were analyzed using CurveExpert ver. 1.4 software.

### Western blot analysis of selected proteins in CAF lysates

Whole-cell lysates were prepared by lysing cells with RIPA buffer containing protease and phosphatase inhibitors for 30 min on ice. The lysates were then frozen in liquid nitrogen, centrifuged at 10 000 × g for 10 min at 4 °C, and stored at − 80 °C. Western blot experiments were performed on 25 μg protein samples using the protocol as described above. The following primary antibodies (with respective dilutions) were used: rabbit anti-idoleamine 1 (IDO1) polyclonal antibody (1:1000), rabbit anti-OPN polyclonal antibody (1:1000), rabbit anti-TGFβ1 polyclonal antibody (1:500) and rabbit anti-TIMP1 polyclonal antibody (1:1000, all from Proteintech, Rosemont, IL, USA). Densitometry analysis was carried out in ImageJ software, with the tested protein levels normalized to β-actin and the respective untreated control (cells untreated with calcitriol).

### Gelatinase activity in CAF lysates

For assessing gelatinase (MMP2 and MMP9) activity, whole-cell extracts were prepared using nondenaturating lysis buffer with 1% (*v/v*) NP-40 (50 mM Tris HCl pH 8, 150 mM NaCl; HIIET PAS, Wroclaw, Poland, Merck, Darmstadt, Germany). Lysates were incubated for 15 min on ice and centrifuged at 16 000 × g for 20 min at 4 °C. Gel electrophoresis was carried out on 25 μg protein samples using homemade gels containing 0.01% gelatin (Sigma‒Aldrich, Saint-Louis, MO, USA). Gels were rinsed with water and incubated with Zymogram Renaturing Buffer (Invitrogen, Waltham, MA, USA) for 20 min at RT twice, followed by washing with water and 30 min of incubation with Zymogram Developing Buffer (Invitrogen, Waltham, MA, USA) at RT. Next, the gels were left overnight with fresh Zymogram Developing Buffer at 37 °C. After a few washes with water, gels were stained with SimplyBlue™ SafeStain (Invitrogen, Waltham, MA, USA) for 1 h at RT. Next, the gels were washed with water twice for one hour each. Gels were photographed using a ChemiDoc Imaging System (Bio-Rad, Hercules, CA, USA). The activity of gelatinases was assessed using a standard densitometry protocol in ImageJ software. The results were normalized to the untreated control (cells untreated with calcitriol).

### Preparation of conditioned media (CM)

A total of 4 × 10^5^ cells/plate were seeded on 10 cm culture plates in triplicate. After 24 h, the medium was replaced with a medium containing calcitriol (0, 1, or 10 ng/mL) for 72 h of incubation. Then, the medium was replaced with a serum-free CAF medium. After 24 h, conditioned medium (CM) was collected, centrifuged (7 min, 350 × g), and stored at − 80 °C. For further experiments, 50% (*v/v*) CM were used.

### Wound healing assay—cancer cells treated with CM from CAFs

To assess the impact of calcitriol-treated CAFs on MCF-7 and MDA-MB-231 cell migration, a wound-healing assay was used. A total of 5 × 10^5^ cancer cells/well were seeded onto 24-well plates. After 24 h, wounds were created by lightly scratching a straight line across the cell monolayers with a 1000 µl plastic pipette tip. After gently washing with medium from the well to remove detached cells, 500 μl of CM was added. Cell images were captured immediately (at time 0 h) and after 3 h. Each experiment was conducted in triplicate. The width of the scratch was measured at five spots in each photo using StreamStart 1.6.1 Software. Migration distance was calculated using the following formula:

$$\text{Distance}=\frac{\mathrm{time}\;0\;\mathrm{scratch}\;\mathrm{width}-\mathrm{time}\;3\;\mathrm{or}\;6\;\mathrm{scratch}\;\mathrm{width}}2$$

### Western blot analysis of selected proteins in lysates of cancer cells treated with CM from CAFs

A total of 1.5 × 10^5^ cells (MCF-7) or 1.7 × 10^5^ cells (MDA-MB-231) were seeded on 6-well plates. After 24 h, the medium was replaced with CAFs CM for 72 h of incubation. Next, whole-cell extracts were prepared using NP-40 lysis buffer following the previously described protocol. Western blot experiments were performed on 20 μg protein samples using the protocol described above. The following primary antibodies (with respective dilutions) were used: rabbit anti-E-cadherin polyclonal antibody (1:5000), rabbit anti-OPN polyclonal antibody (1:1000), and rabbit anti-ZEB1 (zinc finger E-box binding homeobox 1) polyclonal antibody (1:1000), all from Proteintech, Rosemont, IL, USA. Densitometry analysis was carried out in ImageJ software with the tested protein levels normalized to β-actin and the respective untreated control (cancer cells untreated with CM).

### Statistical analysis

The data are expressed as the mean ± standard deviation (SD) or mean ± standard error of the mean (SEM), as described in the figure legends. Statistical analysis was conducted using GraphPad Prism 7.03 (GraphPad Software Inc., USA). Distribution normality was verified using the Shapiro–Wilk test. When comparing two continuous variables, either Student’s *t*-test or the Mann–Whitney U test (if the distribution was not normal) was employed. For comparisons involving three or more variables, one-way ANOVA or the Kruskal–Wallis test (when distribution was not normal) followed by Sidak’s post hoc test was used for multiple comparisons. Differences between groups for which *p* < 0.05 were considered statistically significant.

## Results

Tumor tissue samples from 102 patients were used to isolate CAFs, which were successfully isolated from 91 tumor samples, and 59 primary cultures were established. The number of CAF cultures used in individual experiments along with the corresponding clinical characteristics of the patients are presented in Table 1.

**Table 1:** Clinical characteristics of patients from whom CAFs were isolated. Data are presented separately for each analysis

	**All patients**	**Menopausal status**		**Vitamin D**_**3**_** level**		**Metastases**	
		Premenopausal	Post-menopausal	Deficient	Normal	Absent	Present
	*n* = 127	*n* = 38	*n* = 89	*n* = 89	*n* = 38	*n* = 96	*n* = 31
**Age**	60.6 ± 12.4	47.5 ± 8.6	66.3 ± 9.1^*^	61.8 ± 12.5	57.8 ± 11.9	60.4 ± 12.9	61.5 ± 11.01
**Plasma 25(OH)D**_**3**_** level [ng/ml]**	25.6 ± 12.4	27.6 ± 9.6	24.7 ± 13.4	19.0 ± 6.3	41.0 ± 9.1^#^	25.6 ± 12.7	25.6 ± 11.9
**Plasma FSH level [mIU/ml]**	52.8 ± 35.0	14 ± 16.4	69.5 ± 26.6^*^	53.7 ± 32.6	50.8 ± 40.5	53.3 ± 37.1	51.1 ± 27.8
**Selected protein levels in tumor tissue**	*n* = 80	*n* = 28	*n* = 52	*n* = 57	*n* = 23	*n* = 53	*n* = 27
**Age**	60.2 ± 13.0	46.5 ± 6.6	67.6 ± 8.8^*^	60.4 ± 13.2	59.7 ± 12.7	59.6 ± 13.8	61.3 ± 11.4
**Plasma 25(OH)D**_**3**_** level [ng/ml]**	24.4 ± 11.4	26.5 ± 9.4	23.3 ± 12.2	18.5 ± 6.1	39.0 ± 7.3^#^	24.2 ± 11.0	24.7 ± 12.3
**Plasma FSH level [mIU/ml]**	46.0 ± 33.2	12.6 ± 12.3	64.4 ± 25.8^*^	47.1 ± 31.3	43.5 ± 38.1	45.9 ± 36.2	46.3 ± 26.7
**CAFs’ phenotype**	*n* = 71	*n* = 28	*n* = 43	*n* = 51	*n* = 20	*n* = 52	*n* = 19
**Age**	58.9 ± 13.4	45.4 ± 7.4	67.7 ± 7.9^*^	60.4 ± 13.5	55.1 ± 12.7	58.1 ± 14.0	61.1 ± 11.5
**Plasma 25(OH)D**_**3**_** level [ng/ml]**	25.5 ± 12.9	27.8 ± 9.9	24.1 ± 14.5	18.9 ± 6.2	42.4 ± 9.8^#^	27.1 ± 13.9	21.0 ± 8.4
**Plasma FSH level [mIU/ml]**	48.9 ± 36.5	12.6 ± 12.3	73.0 ± 25.3^*^	47.3 ± 31.0	52.8 ± 48.2	50.2 ± 39.7	45.0 ± 25.5
**CAFs’ sensitivity to antiproliferative calcitriol activity**	*n* = 36	*n* = 15	*n* = 21	*n* = 24	*n* = 12	*n* = 23	*n* = 13
**Age**	58.2 ± 12.6	46.6 ± 7.0	66.5 ± 8.3^*^	60.4 ± 12.4	53.8 ± 12.1	59.5 ± 14.2	55.9 ± 8.9
**Plasma 25(OH)D**_**3**_** level [ng/ml]**	26.4 ± 11.4	28.4 ± 11.6	25 ± 11.3	19.6 ± 5.8	40.2 ± 5.6^#^	27.2 ± 12.7	25.1 ± 8.9
**Plasma FSH level [mIU/ml]**	47.6 ± 32.8	15.1 ± 14.1	71.9 ± 18.2^*^	52.4 ± 31.0	38.3 ± 35.7	47.2 ± 34.7	48.4 ± 30.5
**Level of selected markers on calcitriol-treated CAFs**	*n* = 51	*n* = 18	*n* = 33	*n* = 34	*n* = 17	*n* = 35	*n* = 16
**Age**	59.9 ± 13.6	45.2 ± 7.4	67.9 ± 8.5^*^	61.4 ± 14.1	56.9 ± 12.5	60.6 ± 15.0	59.5 ± 10.4
**Plasma 25(OH)D**_**3**_** level [ng/ml]**	27.1 ± 13.2	27.8 ± 10.7	26.7 ± 14.5	19.5 ± 6.5	42.3 ± 9.3^#^	49.3 ± 34.5	51.2 ± 27.6
**Plasma FSH level [mIU/ml]**	49.9 ± 32.3	13.7 ± 13.3	70.3 ± 19.0^*^	49.2 ± 30.1	51.3 ± 37.1	28.1 ± 14.8	24.9 ± 8.7
**mRNA expression in calcitriol-treated CAFs**	*n* = 44	*n* = 15	*n* = 29	*n* = 29	*n* = 15	*n* = 29	*n* = 15
**Age**	59.8 ± 12.6	46.3 ± 6.9	66.8 ± 8.5^*^	62.4 ± 12.4	54.8 ± 11.7	60.4 ± 13.8	58.7 ± 10.2
**Plasma 25(OH)D**_**3**_** level [ng/ml]**	27.6 ± 13.6	29.3 ± 11.0	26.7 ± 14.9	19.3 ± 5.9	43.6 ± 9.1^#^	29.3 ± 15.5	24.2 ± 8.7
**Plasma FSH level [mIU/ml]**	52.9 ± 31.9	15.4 ± 13.9	73.0 ± 16.8^*^	53.5 ± 28.3	51.7 ± 38.7	54.4 ± 33.9	49.8 ± 28.1
**Protein expression in calcitriol-treated CAFs**	*n* = 35	*n* = 12	*n* = 23	*n* = 21	*n* = 14	*n* = 21	*n* = 14
**Age**	59.7 ± 13.1	45.4 ± 7.3	67.2 ± 8.4^*^	62.4 ± 12.9	55.7 ± 12.9	59.4 ± 14.3	60.3 ± 11.1
**Plasma 25(OH)D**_**3**_** level [ng/ml]**	28.4 ± 12.7	29.3 ± 12.3	27.9 ± 13.2	19.5 ± 6.1	41.9 ± 6.5^#^	30.0 ± 14.2	25.5 ± 9.3
**Plasma FSH level [mIU/ml]**	53.4 ± 32.9	14.2 ± 15.5	74.8 ± 14.9^*^	53.8 ± 29.2	52.8 ± 38.8	53.8 ± 34.6	52.6 ± 26.7
**Protein concentration in cell supernatants from calcitriol-treated CAFs**	*n* = 51	*n* = 18	*n* = 33	*n* = 34	*n* = 17	*n* = 35	*n* = 16
**Age**	59.9 ± 13.6	45.2 ± 7.4	67.9 ± 8.5^*^	61.4 ± 14.1	59.6 ± 12.5	60.1 ± 15.0	59.5 ± 10.4
**Plasma 25(OH)D**_**3**_** level [ng/ml]**	27.1 ± 13.2	27.8 ± 10.7	26.7 ± 14.5	19.5 ± 6.5	42.3 ± 9.3^#^	28.1 ± 14.8	24.9 ± 8.7
**Plasma FSH level [mIU/ml]**	49.9 ± 32.3	13.7 ± 13.3	70.3 ± 19.0^*^	49.2 ± 30.1	51.3 ± 37.1	49.3 ± 34.5	51.2 ± 28.6
**Gelatinases activity in calcitriol-treated CAFs**	*n* = 16	*n* = 10	*n* = 6	*n* = 9	*n* = 7	*n* = 10	*n* = 6
**Age**	54.8 ± 12.7	46.6 ± 6.9	68.3 ± 6.6^*^	58.11 ± 9.6	50.4 ± 15.6	50.7 ± 13.6	61.5 ± 8.1
**Plasma 25(OH)D**_**3**_** level [ng/ml]**	29.3 ± 12.2	31.3 ± 12.8	26.0 ± 11.5	20.0 ± 6.9	41.2 ± 3.8^#^	31.1 ± 13.1	26.3 ± 11.2
**Plasma FSH level [mIU/ml]**	34.2 ± 32.4	14.9 ± 15.6	72.7 ± 18.4^*^	41.3 ± 27.2	26.0 ± 38.0	28.8 ± 34.7	42.2 ± 29.7
**Calcitriol-treated CAFs’ impact on breast cancer cells**	*n* = 16	*n* = 7	*n* = 9	*n* = 10	*n* = 6	*n* = 9	*n* = 7
**Age**	60.7 ± 11.7	49.1 ± 5.0	69.7 ± 5.6^*^	59.5 ± 10.1	62.7 ± 14.9	59.9 ± 14.0	63.0 ± 8.4
**Plasma 25(OH)D**_**3**_** level [ng/ml]**	26.9 ± 10.4	26.4 ± 12.3	27.2 ± 9.5	20.4 ± 6.6	37.6 ± 5.1^#^	26.6 ± 11.0	27.3 ± 10.5
**Plasma FSH level [mIU/ml]**	45.4 ± 32.2	19.1 ± 17.1	68.4 ± 22.9^*^	46.5 ± 29.9	43.8 ± 38.4	40.5 ± 36.6	52.7 ± 25.7

Patients were classified into groups according to plasma 25(OH)D_3_ levels (< 30 ng/mL—deficient, > 30 ng/mL—normal), plasma FSH levels (< 25.8 mIU/mL—premenopausal, > 25.8 mIU/mL—postmenopausal) and the presence of regional or distant metastases (if any—present, otherwise absent). Data are presented as the mean ± SD. Statistical analysis was carried out using Student’s *t*-test or the Mann‒Whitney U test: ^*^p < 0.05—premenopausal vs postmenopausal, ^#^*p* < 0.05—normal vs deficient vitamin D_3_ level

### Tumors from patients with varying metastatic statuses or different 25(OH)D_3_ statuses exhibit differential expression of TGFβ, β-catenin and CYP24A1

**Fig. 1:**
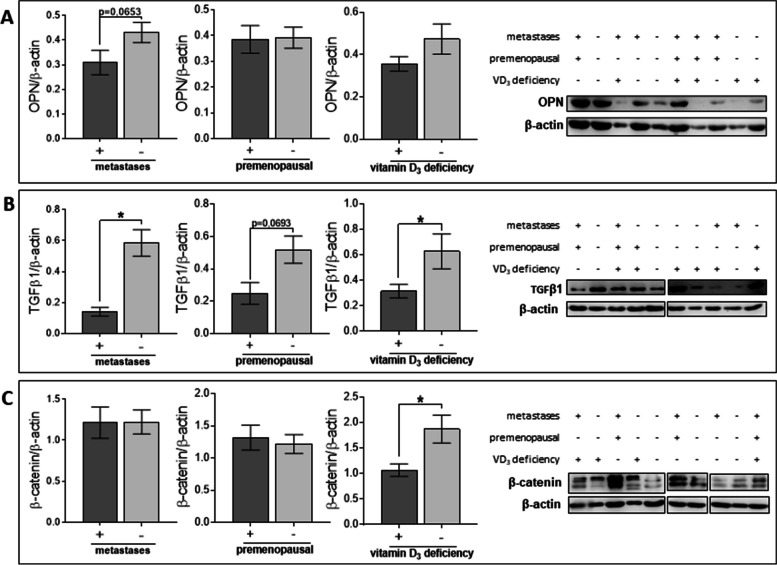
OPN, TGFβ, and β-catenin levels in tumor tissues from patients with different clinical characteristics. **A** Comparison of OPN (osteopontin) levels in tumors from patients with different clinical characteristics. **B** Comparison of TGFβ1 (transforming growth factor β) levels in tumors from patients with different clinical characteristics. **C** Comparison of β-catenin levels in tumors from patients with different clinical characteristics. Representative cropped blots of tumors from 10 patients are shown next to the graphs. For TGFβ1 and β-catenin bonds from different part of the same blot and different blots were combined. Molecular weight of analyzed proteins: OPN—40 kDa, TGFβ1—35 kDa, β-catenin—92 kDa. Full-length blots are presented in Figure S2 in the Supplementary Materials. Patients were classified into groups according to plasma 25(OH)D_3_ levels (VD3, < 30 ng/mL—deficiency (*n* = 57), > 30 ng/mL—normal (*n* = 22)), plasma FSH levels (< 25.8 mIU/mL—premenopausal (*n* = 28), > 25.8 mIU/mL—postmenopausal (*n* = 52)) and regional or distant metastasis presence (if any—present (*n* = 23), otherwise absent (*n* = 57)). Densitometric analysis was performed using ImageJ software. The results were normalized to β-actin levels. Data are presented as the mean ± SD. Statistical analysis was carried out using Student’s *t*-test or the Mann–Whitney U test. *p ≤ 0.05

First, we decided to assess whether the expression of proteins involved in tumor progression and vitamin D_3_ signaling or metabolism differed in tumors from patients with different clinical characteristics. No difference in OPN levels was observed between tumors from patients with different clinical characteristics. Only a tendency (*p* = 0.0653) toward higher OPN levels in nonmetastatic tumors was observed (Fig. 1A). TGFβ levels were higher in tumors from nonmetastatic patients compared to those from metastatic patients, and a similar tendency (*p* = 0.0693) was noted between postmenopausal and premenopausal patients (Fig. 1B). Moreover, tumors from patients with normal plasma 25(OH)D_3_ levels were characterized by higher TGFβ and β-catenin levels than tumors from vitamin D_3_-deficient patients (Fig. 1B–C).

There were no differences in VDR or CYP27B1 levels between tumors from patients with different clinical characteristics (Figure S1A–B in Supplementary Materials). However, CYP24A1 levels were lower in tumors from patients with normal plasma 25(OH)D_3_ levels (Figure S1C in Supplementary Materials).

### CAF infiltration in tumor tissues

**Fig. 2:**
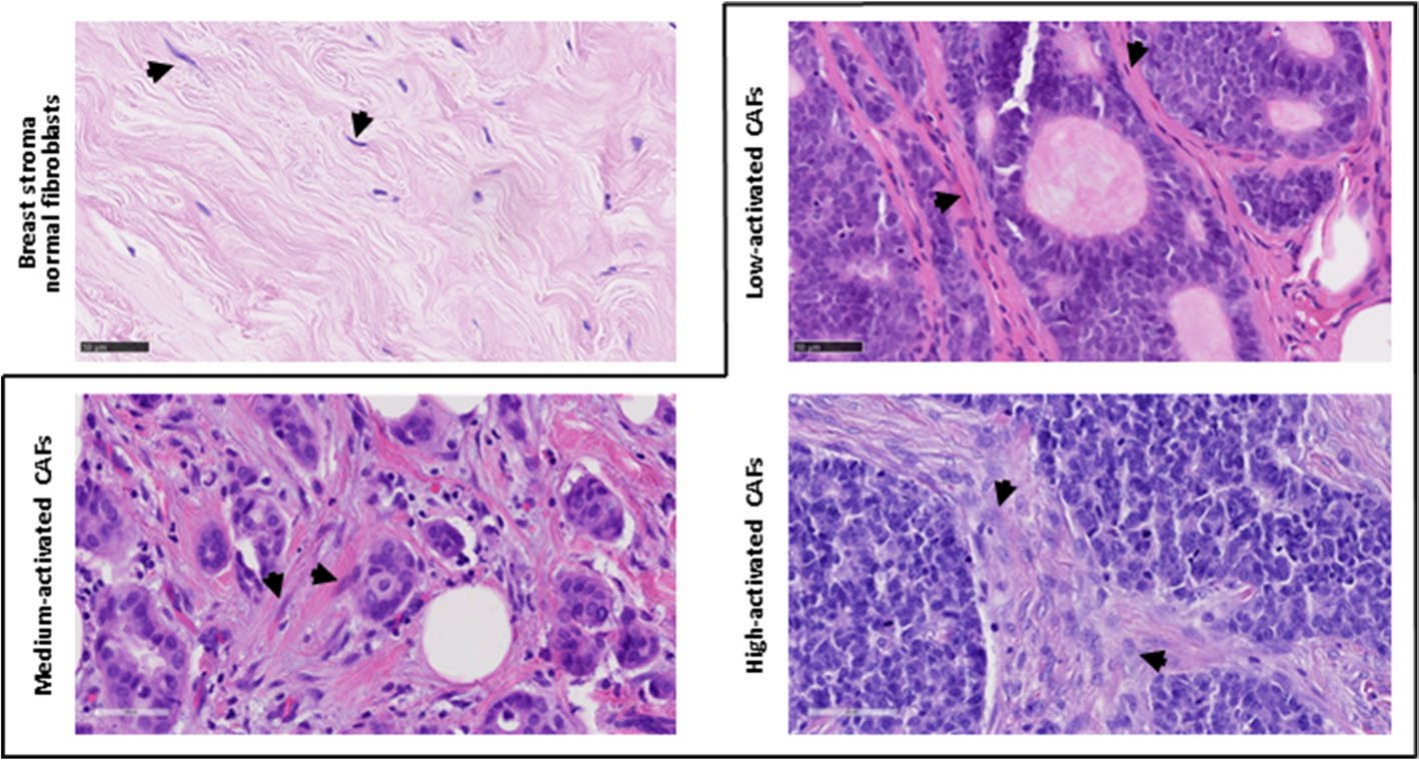
Representative images of CAFs with various activation levels. Arrows point to examples of fibroblasts

Fibroblast density and activation levels were assessed in 106 cases. Forty-five tumor tissues were characterized by a low CAF infiltration score, 21 by medium–low, 17 by medium, 15 by medium–high, and 8 by high CAF infiltration. No differences were found in the fibroblast infiltration score among tumor tissues derived from patients with different clinical characteristics (Table S4 in Supplementary Materials). Moreover, the CAF infiltration score was not correlated with tumor grade (*r* =  − 0.0081, CI: − 0.2069 to 0.1913). Representative images of mammary normal fibroblasts and CAFs with various activation levels are presented in Fig. 2.

### CAF infiltration status is associated with CYP24A1, CYP27B1, and OPN tumor tissue levels

Moreover, CAF activation, density, or infiltration were not found to be associated with VDR tumoral levels. Activation of CAFs was negatively correlated with the tumoral level of CYP27B1 (Table 2), while CAF density and CAF infiltration were inversely correlated with CYP24A1 levels and positively correlated with OPN levels. These associations were observed when fibroblasts from all tissue samples were assessed and for tumor tissues derived from vitamin D_3_-deficient patients (Table 2). However, no additional correlations between the fibroblast infiltration score and the levels of CYP24A1, OPN, TGFβ1, and β-catenin were found (Table 2).

**Table 2:** Spearman correlation analysis of CAF statuses and tumor tissue levels of selected proteins

	VDR level	CYP24A1 level	CYP27B1 level	OPN level	TGFβ1 level	β-catenin level
CAF activation and protein levels						
All patients	0.11 (− 0.35 to 0.14)	− 0.016 (− 0.39 to 0.80)	− 0.25 (− 0.47 to − 0.004)^*^	0.098 (− 0.14 to 0.33)	− 0.1479 (− 0.40 to 0.13)	− 0.20 (− 0.44 to 0.61)
CAF density and protein levels						
All patients	− 0.66 (− 0.31 to 0.19)	− 0.25 (− 0.46 to − 0.000)^*^	0.021 (− 0.23 to 0.27)	0.29 (0.047 to 0.50)^*^	0.013 (− 0.26 to 0.29)	− 0.060 (− 0.32 to 0.21)
CAF infiltration and protein levels						
All patients	− 0.17 (− 0.41 to 0.082)	− 0.25 (− 0.46 to − 0.005)^*^	− 0.12 (− 0.36 to 0.14)	0.25 (0.009 to 0.46)^*^	− 0.079 (− 0.34 to 0.20)	− 0.15 (− 0.39 to 0.11)
Premenopausal	− 0.42 (− 073 to 0.041)	− 0.11 (− 0.54 to 0.36)	− 0.30 (− 0.66 to 0.018)	0.22 (− 0.25 to 0.60)	0.00 − 0.52 to 0.60)	− 0.018 (− 0.48 to 0.45)
Postmenopausal	− 0.048 (− 0.35 to 0.26)	− 0.23 (− 0.49 to 0.068)	− 0.095 (− 0.39 to 0.22)	0.26 (− 0.03 to 0.52)	0.052 (− 0.28 to 0.37)	− 0.25 (− 0.53 to 0.077)
Vitamin D_3_—deficient	− 0.1281 (− 0.41 to 0.18)	− 0.38 (− 0.60 to − 0.10)^**^	− 0.060 (− 0.35 to 0.24)	0.40 (0.13 to 0.61)^**^	− 0.025 (− 0.35 to 0.30)	− 0.049 (− 0.35 to 0.26)
Vitamin D_3_—normal	− 0.31 (− 0.67 to 0.16)	− 0.29 (− 0.69 to 0.23)	− 0.21 (− 0.61 to 0.29)	− 0.054 (− 0.50 to 0.41)	− 0.14 (− 0.62 to 0.41)	− 0.20 (− 0.62 to 0.31)
Metastases present	− 0.24 (− 0.61 to 0.22)	− 0.031 (− 0.43 to 0.38)	0.015 (− 0.40 to 0.43)	0.32 − 0.098 to 0.64)	− 0.25 (− 0.66 to 0.28)	− 0.048 (− 0.48 to 0.40)
Metastases absent	− 0.19 (− 0.47 to 0.13)	− 0.27 (− 0.53 to 0.039)	− 0.13 (− 0.43 to 0.19)	0.23 (− 0.077 to 0.50)	− 0.021 (− 0.36 to 0.32)	− 0.17 (− 0.47 to 0.16)

Data are presented as the *r* Spearman coefficient and 95% confidence interval. Statistical analysis was carried out using nonparametric Spearman correlation: ^*^p ≤ 0.05, ^**^p ≤ 0.01

### Isolation of CAFs

CAFs were isolated from 91 tumor specimens, yielding varying numbers of cells per isolation, ranging from 6 × 10^3^ to 1.01 × 10^6^ cells. Phenotype characterization was carried out on 71 freshly isolated CAFs. Cells that tested negative for epithelial (EpCAM), endothelial (CD31), or immune (CD45) markers were classified as CAFs (the gating strategy is presented in Figure S4 in Supplementary Materials). Among the living and single cells, on average, 19% were EpCAM^+^, 4% were CD31^+^, and 15% were CD45^+^.

### Tumors from patients with different clinical characteristics generate CAFs with similar phenotypes

Isolated CAFs were positive for selected markers (αSMA, PDPN, PDGFRβ, TNC, and FSP1). However, no differences were observed in the levels of these proteins between CAFs isolated from patients with different clinical characteristics (vitamin D_3_ status, menopausal status, or presence of metastases) (Figure S5A1–A5 in Supplementary Materials). Additionally, isolated CAFs were positive for vimentin (Figure S5B in Supplementary Materials).

### CAFs are sensitive to calcitriol antiproliferative activity

**Fig. 3:**
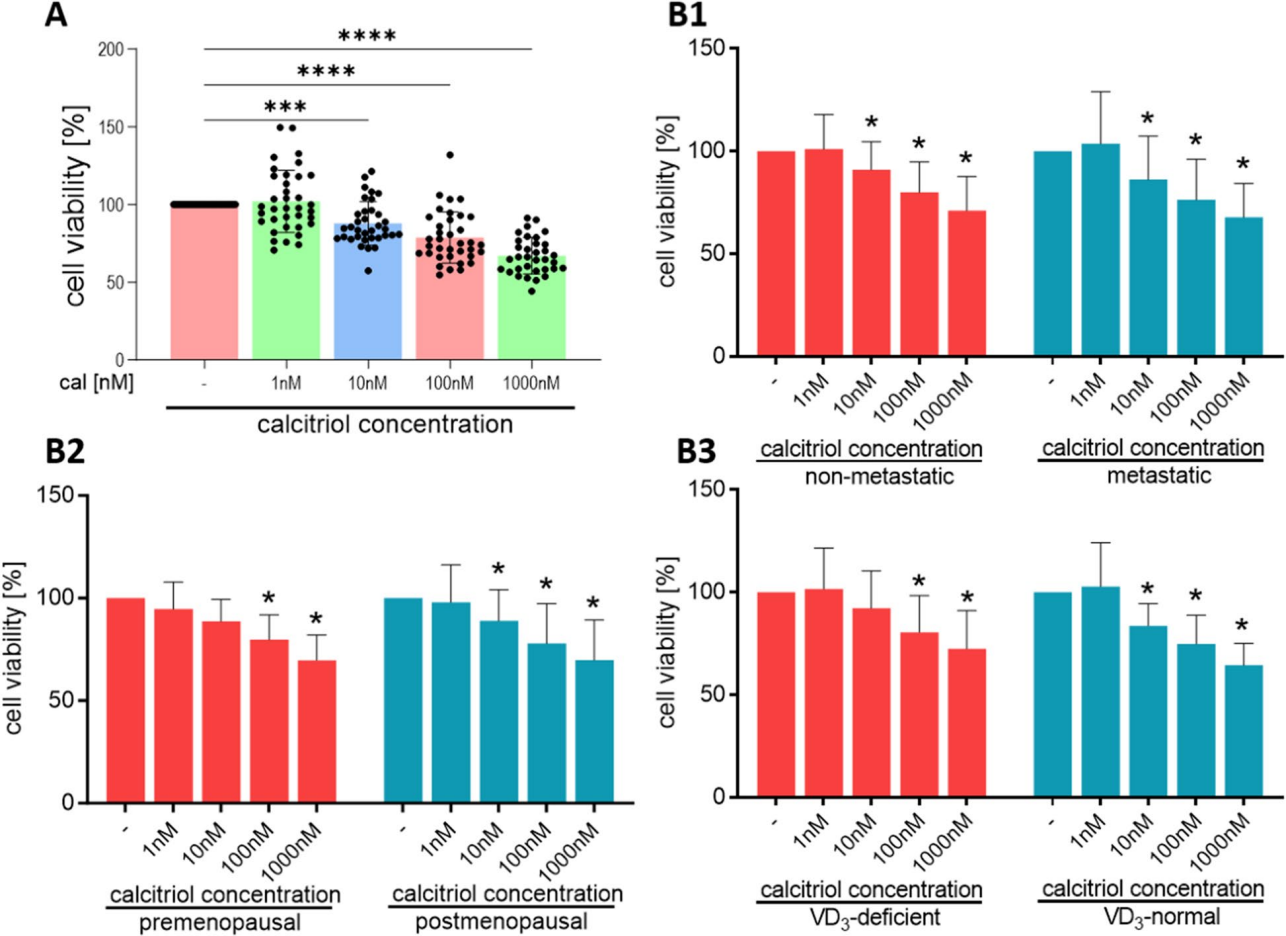
CAFs viability after calcitriol treatment. **A** data presented for CAFs from all patients. **B1** data presented for CAFs derived from metastatic (*n* = 13) or nonmetastatic tumors (*n* = 23). **B2** data presented for CAFs derived from tumors from pre- (*n* = 15) or postmenopausal patients (*n* = 21). **B3** data presented for CAFs derived from tumors of patients with deficient (*n* = 24) or normal plasma 25(OH)D_3_ levels (*n* = 12). CAFs were stimulated with different calcitriol concentrations (1 nM, 10 nM, 100 nM, or 1000 nM) for 72 h. Patients were classified into groups according to plasma 25(OH)D_3_ levels (VD3, < 30 ng/mL—deficient, > 30 ng/mL—normal), plasma FSH levels (< 25.8 mIU/mL—premenopausal, > 25.8 mIU/mL—postmenopausal) and regional or distant metastasis presence (if any—metastatic, otherwise nonmetastatic). Viability was calculated as a percentage of untreated cells. Data are presented as the mean ± SD. Statistical analysis was carried out using Student’s *t*-test or the Mann‒Whitney U test for single comparisons and one-way ANOVA for multiple comparisons. *p ≤ 0.05 compared to untreated cells. *p ≤ 0.05, **p ≤ 0.01, ***p ≤ 0.001, ****p ≤ 0.001

Calcitriol treatment diminished CAFs’ viability when using concentrations of 10 nM to 1000 nM calcitriol (Fig. 3A and B1-B3). For CAFs isolated from premenopausal or vitamin D_3_-deficient patients, calcitriol’s antiproliferative activity was observed only after treatment with 100 nM and 1000 nM calcitriol (Fig. 3B2–B3).

### Calcitriol does not affect the CAF phenotype

**Fig. 4:**
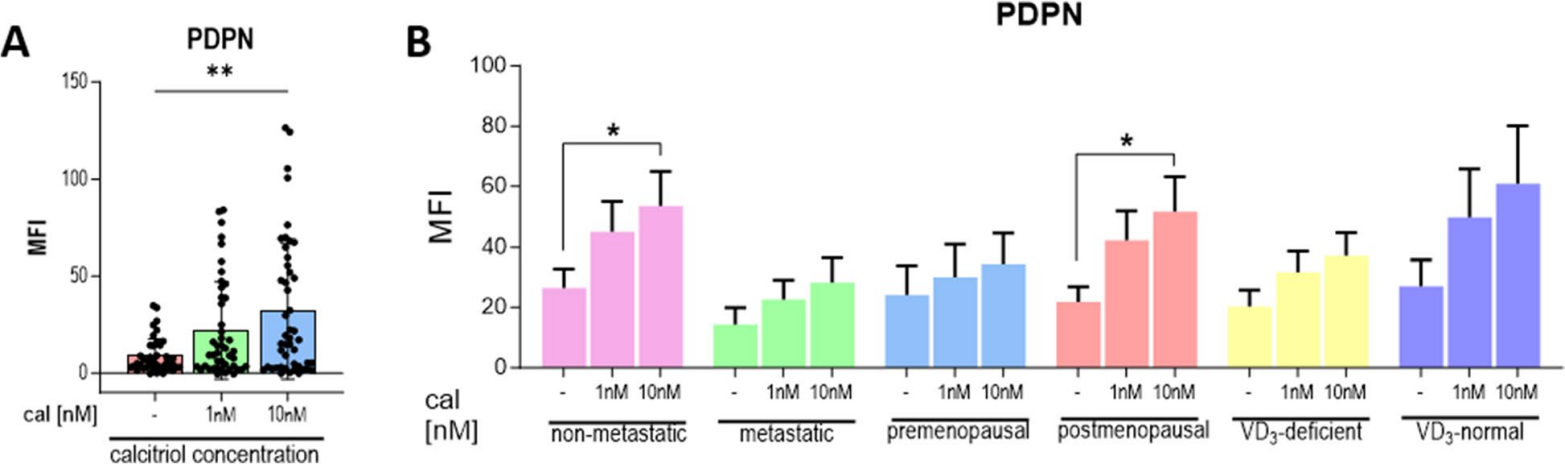
Calcitriol impact on the phenotype of CAFs from tumors of patients with different clinical characteristics. Levels of PDPN (podoplanin) were assessed: **A** data presented for CAFs from all patients, **B** data presented for CAFs from tumors of patients with different clinical characteristics. Other protein levels are presented in Figure S6 in the Supplementary Materials. CAFs were treated with calcitriol (cal; 1 nM or 10 nM) for 72 h. Patients were classified into groups according to plasma 25(OH)D_3_ levels (VD3, < 30 ng/mL—deficient (*n* = 34), > 30 ng/mL—normal (*n* = 17)), plasma FSH levels (< 25.8 mIU/mL—premenopausal (*n* = 18), > 25.8 mIU/mL—postmenopausal (*n* = 33)) and regional or distant metastasis presence (if any—metastatic (*n* = 16), otherwise nonmetastatic (*n* = 35)). Protein levels are presented as median fluorescence (MFI) normalized to the untreated control. Data are presented as the mean ± SEM. Statistical analysis was carried out using Student’s *t*-test or the Mann‒Whitney U test for single comparisons and one-way ANOVA or the Kruskal‒Wallis test for multiple comparisons. *p ≤ 0.05. **p ≤ 0.01

Calcitriol treatment did not change the levels of αSMA, PDGFRβ, TNC, or FSP1 (Figure S4A–D). Only PDPN levels increased significantly in CAFs derived from tumors of nonmetastatic or postmenopausal patients (Fig. 4A-B).

### Identification of targets for further evaluation

First, screening q-PCR was performed. For the majority of analyzed CAF cultures (16/19), calcitriol treatment induced *CYP24* expression and downregulated *VDR* expression (14/19) (expression matrix presented in Figure S7 in the Supplementary Materials). A wide panel of genes (Table S3 in Supplementary Materials) allowed the identification of targets for further evaluation. The following genes and proteins were chosen: *CCL2*, *MMP9*, *PDPN*, *TIMP1*, *TNC*, *SPP1*, and *VDR* for mRNA expression assessment using RT‒PCR; CCL2, CXCL12, HGF, MMP9, TNC, and OPN for the assessment of secreted protein concentrations using ELISA kits; and IDO1, MMP2, MMP9, TIMP1, TGFβ1 and OPN for the analysis of protein levels using western blot or gelatin zymography assays.

### Calcitriol regulates the mRNA expression of selected genes in CAFs

**Fig. 5:**
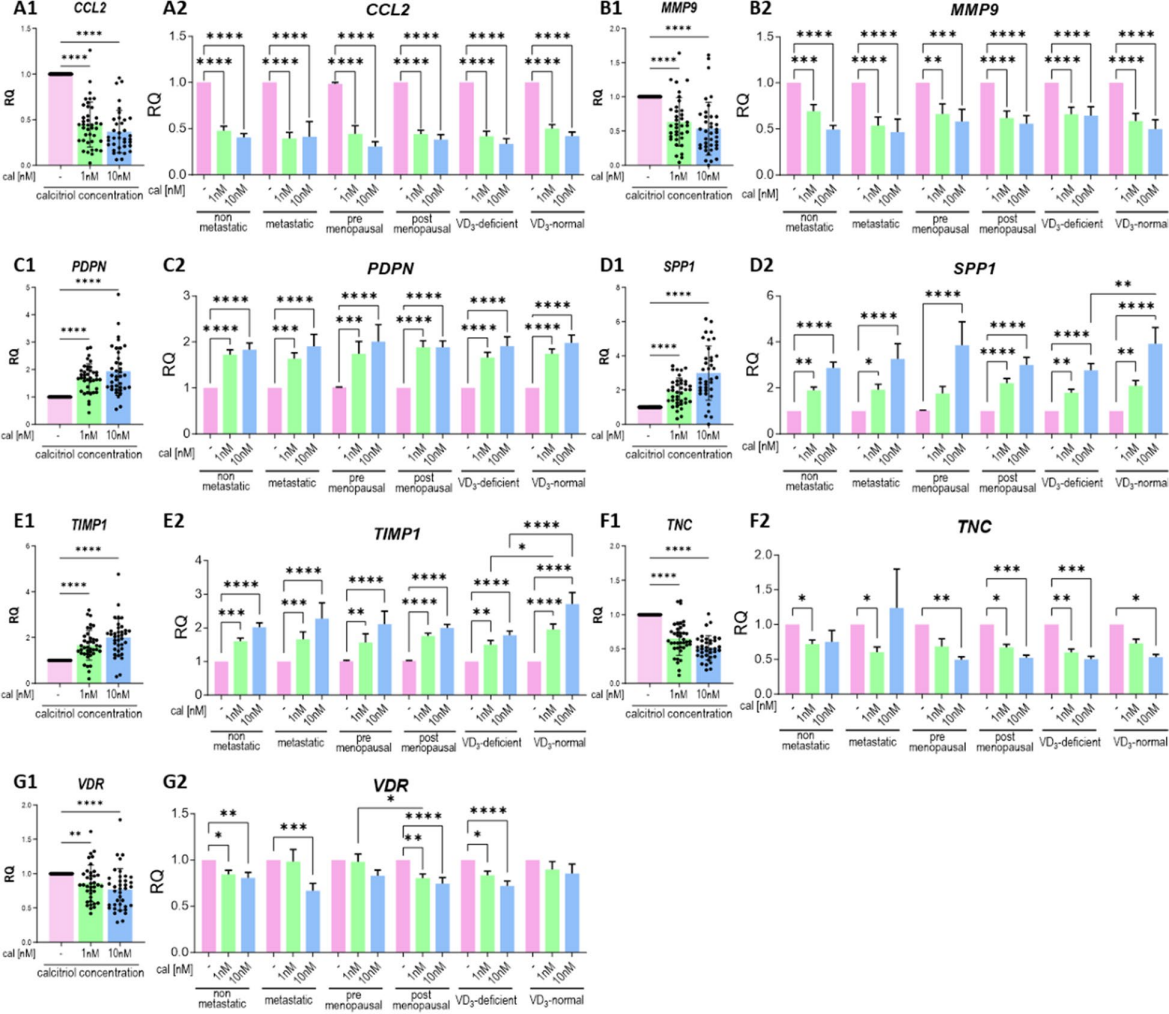
Selected mRNA expression in calcitriol-treated CAFs derived from tumors of patients with different clinical characteristics. CAFs were treated with calcitriol (cal; 1 nM or 10 nM) for 72 h. **A1**–**G1** data presented for CAFs from all patients. **A2**–**G2** data presented for CAFs derived from tumors of patients with different clinical characteristics. **A**–**C**—C motif chemokine ligand 2 (CCL2), **B** metalloproteinase 9 (MMP9), podoplanin (PDPN), secreted phosphoprotein 1 (SPP1), tissue metalloproteinases inhibitor 1 (TIMP1), tenascin C (TNC), vitamin D receptor (VDR). Patients were classified into groups according to plasma 25(OH)D_3_ levels (VD3, < 30 ng/mL—deficient (*n* = 29), > 30 ng/mL—normal (*n* = 15)), plasma FSH levels (< 25.8 mIU/mL—premenopausal (*n* = 15), > 25.8 mIU/mL—postmenopausal (*n* = 29)) and regional or distant metastasis presence (if any—metastatic (*n* = 15), otherwise nonmetastatic (*n* = 29)). mRNA expression was calculated according to the ΔΔCt comparative method and normalized to the untreated control. Data are presented as the mean ± SEM. Statistical analysis was carried out using Student’s *t*-test or the Mann‒Whitney U test for single comparisons and one-way ANOVA or the Kruskal–Wallis test for multiple comparisons. *p ≤ 0.05, **p ≤ 0.01, ***p ≤ 0.001, ****p ≤ 0.001

**Fig. 6:**
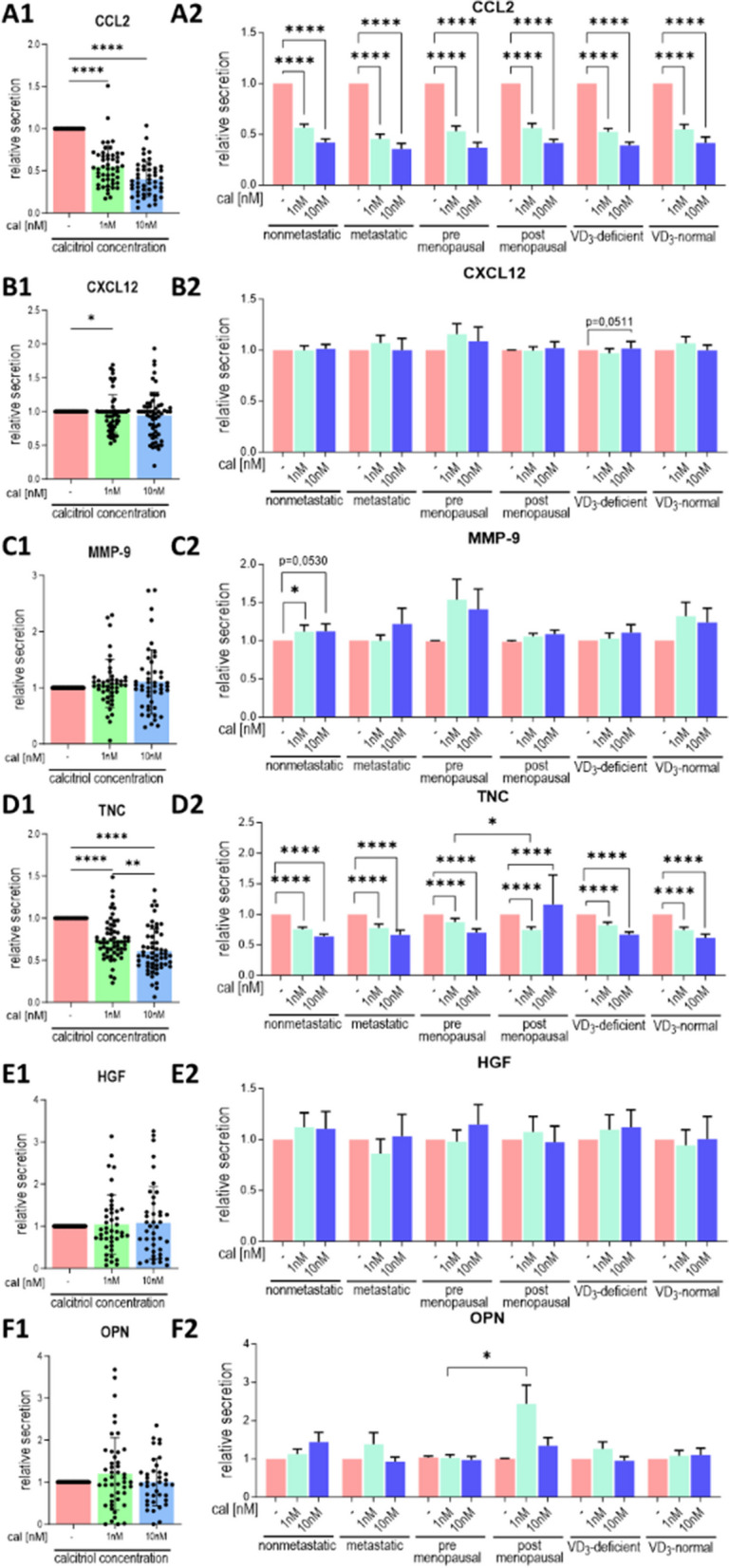
Selected molecules secreted from calcitriol-treated CAFs derived from tumors of patients with different clinical characteristics. **A1**–**F1** data presented for CAFs from all patients. **A2**–**F2** data presented for CAFs derived from tumors of patients with different clinical characteristics. CAFs were treated with calcitriol (cal; 1 nM or 10 nM) for 72 h. **A**–**C**–C motif chemokine ligand 2 (CCL2), **B**-**C**-X-C motif chemokine ligand 12 (CXCL12), **C** tenascin C (TNC), **D** metalloproteinase 9 (MMP9), **E** hepatocyte growth factor (HGF), **F** osteopontin (OPN). Patients were classified into groups according to plasma 25(OH)D_3_ levels (VD_3_, < 30 ng/mL—deficient (*n* = 34), > 30 ng/mL—normal (*n* = 17)), plasma FSH levels (< 25.8 mIU/mL—premenopausal (*n* = 18), > 25.8 mIU/mL—postmenopausal (*n* = 33)) and regional or distant metastasis presence (if any—metastatic (*n* = 16), otherwise nonmetastatic (*n* = 35)). The concentration of secreted protein was calculated based on a standard curve using CurveExpert 1.4. The results were normalized to the untreated control. Data are presented as the mean ± SEM. Statistical analysis was carried out using Student’s *t*-test or the Mann‒Whitney U test for single comparisons and one-way ANOVA or the Kruskal‒Wallis test for multiple comparisons. *p ≤ 0.05, **p ≤ 0.01, ***p ≤ 0.001, ****p ≤ 0.001

Calcitriol treatment decreased the mRNA expression of *CCL2* and *MMP9* (Fig. 5A–B) and increased the expression of *PDPN*, *SPP1* (encoding OPN), and *TIMP1* in CAFs derived from tumors of patients with different clinical characteristics (Fig. 5C–E). Moreover, in CAFs derived from tumors of vitamin D_3_-normal patients, calcitriol treatment resulted in a greater increase in *SPP1* and *TIMP1* expressions than in CAFs derived from vitamin D_3_-deficient patients (Fig. 5D2 and E3). Calcitriol treatment reduced the expression of TNC and VDR (Fig. 5F and G); however, there was no effect on VDR expression when CAFs from tumors of vitamin D_3_-normal patients were treated with calcitriol (Fig. 6G2).

### Calcitriol regulates the secretion of selected proteins by CAFs

**Fig. 7:**
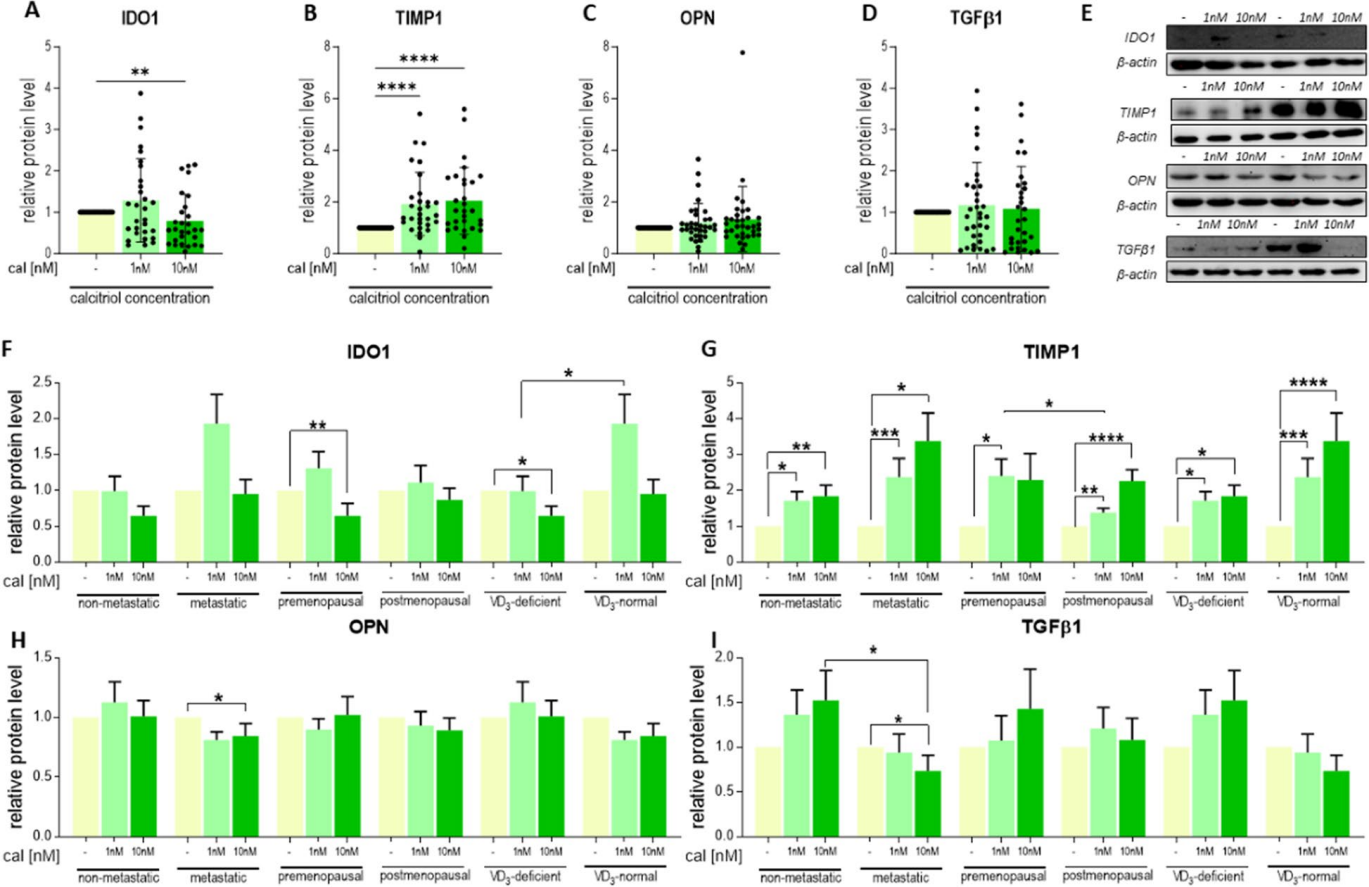
Selected protein levels in calcitriol-treated CAFs derived from tumors of patients with different clinical characteristics. **A**–**D** data presented for CAFs from all patients. **E** representative cropped blots. **F**–**I** data presented for CAFs derived from tumors of patients with different clinical characteristics. **A** and **F** idoleamine 1 (IDO1), **B** and **G** tissue metalloproteinase inhibitor 1 (TIMP1), **C** and **H** osteopontin (OPN), **D** and **I** transforming growth factor β (TGFβ). CAFs were treated with calcitriol (cal; 1 nM or 10 nM) for 72 h. The molecular weights of the analyzed proteins were as follows: IDO1—40 kDa, OPN—40 kDa, TIMP1—28 kDa, and TGFβ1—35 kDa. Full-length blots are presented in Figure S8 in the Supplementary Materials. Patients were classified into groups according to plasma 25(OH)D_3_ levels (VD3, < 30 ng/mL—deficient (*n* = 21), > 30 ng/mL—normal (*n* = 14)), plasma FSH levels (< 25.8 mIU/mL—premenopausal (*n* = 12), > 25.8 mIU/mL—postmenopausal (*n* = 23)) and regional or distant metastasis presence (if any—metastatic (*n* = 14), otherwise nonmetastatic (*n* = 21)). Densitometric analysis was performed using ImageJ software. The results were normalized to β-actin levels and untreated controls. Data are presented as the mean ± SEM. Statistical analysis was carried out using Student’s *t*-test or the Mann‒Whitney U test for single comparisons and one-way ANOVA or the Kruskal‒Wallis test for multiple comparisons. *p ≤ 0.05, **p ≤ 0.01, ***p ≤ 0.001, ****p ≤ 0.001

Calcitriol treatment resulted in decreased secretion of CCL2, CXCL12, or TNC into the culture medium and increased secretion of MMP-9 but did not affect the secretion of HGF or OPN (Fig. 6A–F). A reduction in CCL2 production was observed in CAFs from patients with various clinical characteristics following calcitriol treatment (Fig. 6A1–A2). Secretion of CXCL2 was diminished only when CAFs from all tumors were assessed (Fig. 6B1). There was also a nonsignificant decrease in CXCL12 production in CAFs from tumors of patients with vitamin D_3_ deficiency (*p* = 0.0511) (Fig. 6B2). Despite a significant decrease in TNC production in all CAFs included in this analysis (Fig. 6C1), TNC production increased in 10 nM calcitriol-treated CAFs from tumors of postmenopausal patients (Fig. 6C2). In CAFs from other origins, calcitriol decreased TNC secretion (Fig. 6C2). Furthermore, calcitriol treatment increased MMP-9 production only in CAFs from nonmetastatic patients (Fig. 6D2). Moreover, 10 nM calcitriol treatment reduced IDO1 levels in CAFs from tumors of all patients (Fig. 7A and E) and in CAFs from tumors of premenopausal patients or those with vitamin D_3_ Deficiency (Fig. 7F). Similar to mRNA levels, the TIMP1 protein level also increased after calcitriol treatment in CAFs derived from tumors of patients with different clinical characteristics (Fig. 7B, E and G). Calcitriol reduced OPN levels only in CAFs derived from the tumors of metastatic patients (Fig. 7C, E and H). Similar to OPN, the level of TGFβ1 was reduced only in CAFs from tumors of metastatic patients (Fig. 7D, E and I).

### Calcitriol reduces gelatinase activity in CAFs

**Fig. 8:**
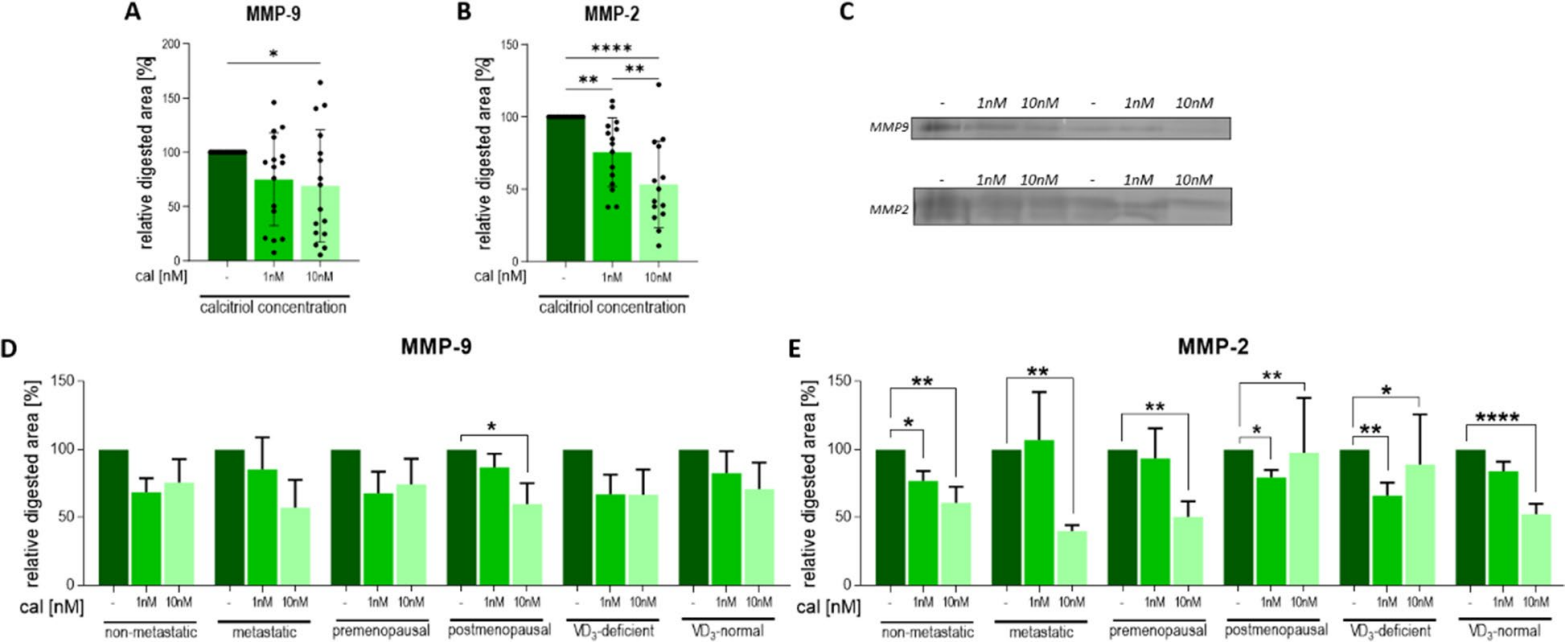
Gelatinase activity in calcitriol-treated CAFs derived from tumors of patients with different clinical characteristics. **A**–B data presented for CAFs from all patients. **D**–**E** Data presented for CAFs derived from tumors of patients with different clinical characteristics. CAFs were treated with calcitriol (cal; 1 nM or 10 nM) for 72 h. **C** representative cropped gels, negative photography. **A** and **D** MMP-2 activity, **B** and **E** MMP-9 activity, Molecular weight of analyzed proteins: MMP2—55–66 kDa, MMP9—97 kDa. Full-length gels are presented in Figure S9 in the Supplementary Materials. Patients were classified into groups according to plasma 25(OH)D_3_ levels (VD3, < 30 ng/mL—deficient (*n* = 9), > 30 ng/mL—normal (*n* = 7)), plasma FSH levels (< 25.8 mIU/mL—premenopausal (*n* = 6), > 25.8 mIU/mL—postmenopausal (*n* = 10)) and regional or distant metastasis presence (if any—metastatic (*n* = 6), otherwise nonmetastatic (*n* = 10)). Densitometric analysis was performed using ImageJ software. The results are presented as the percentage of untreated cells. Data are presented as the mean ± SEM. Statistical analysis was carried out using Student’s *t*-test or the Mann‒Whitney U test for single comparisons and one-way ANOVA or the Kruskal‒Wallis test for multiple comparisons. *p ≤ 0.05

Upon assessing CAFs from all patients, it was evident that calcitriol reduced MMP-9 and MMP-2 activity (Fig. 8A-C). However, when CAFs from patients with differing clinical characteristics were analyzed, MMP-9 activity was reduced only in CAFs from postmenopausal patients’ tumors (Fig. 8D), whereas MMP-2 activity was reduced in each group (Fig. 8E).

### Calcitriol modulates the impact of CAFs on breast cancer cells

**Fig. 9:**
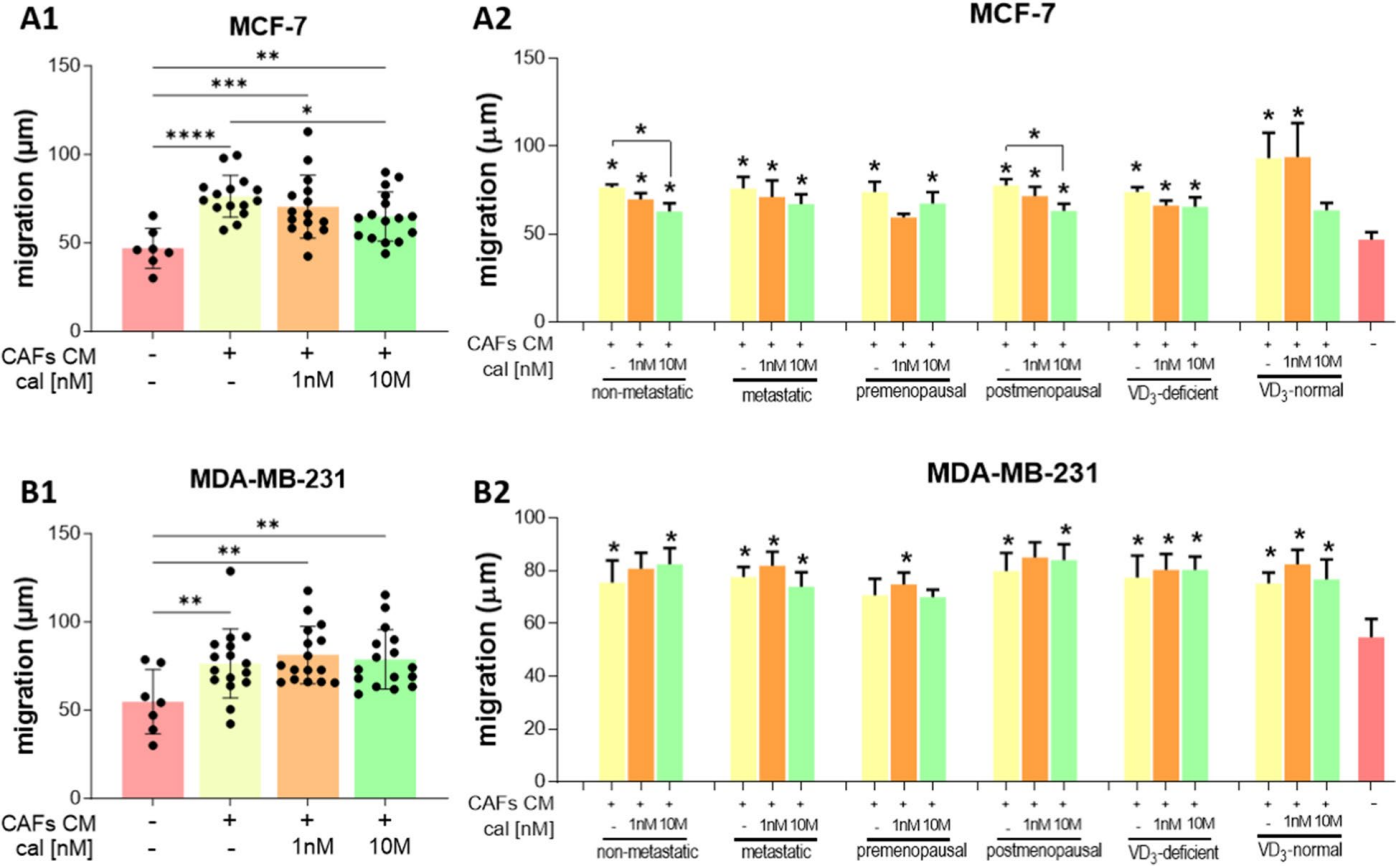
Impact of CAFs on breast cancer cell migration. CAFs were derived from the tumors of patients with different clinical characteristics. **A** MCF-7 cells, **B** MDA-MB-231 cells. CAFs were stimulated with calcitriol (cal) for 72 h prior to CM generation for 24 h. CM was applied to cancer cells immediately after wound generation. Cells were photographed at 0 and 3 h after CM application using Stream Start 1.6.1 software. Patients were classified into groups according to plasma 25(OH)D_3_ levels (VD_3_, < 30 ng/mL—deficient (*n* = 10), > 30 ng/mL—normal (*n* = 6)), plasma FSH levels (< 25.8 mIU/mL—premenopausal (*n* = 7), > 25.8 mIU/mL—postmenopausal (*n* = 9)) and regional or distant metastasis presence (if any—metastatic (*n* = 7), otherwise nonmetastatic (*n* = 9)). Data are presented as the mean migration distance ± SEM. Statistical analysis was carried out using Student’s *t*-test or Mann‒Whitney U test for single comparisons and one-way ANOVA or Kruskal‒Wallis tests for multiple comparisons. *p ≤ 0.05 compared to untreated cancer cells. *p ≤ 0.05, **p ≤ 0.01, ***p ≤ 0.001, ****p ≤ 0.001

**Fig. 10:**
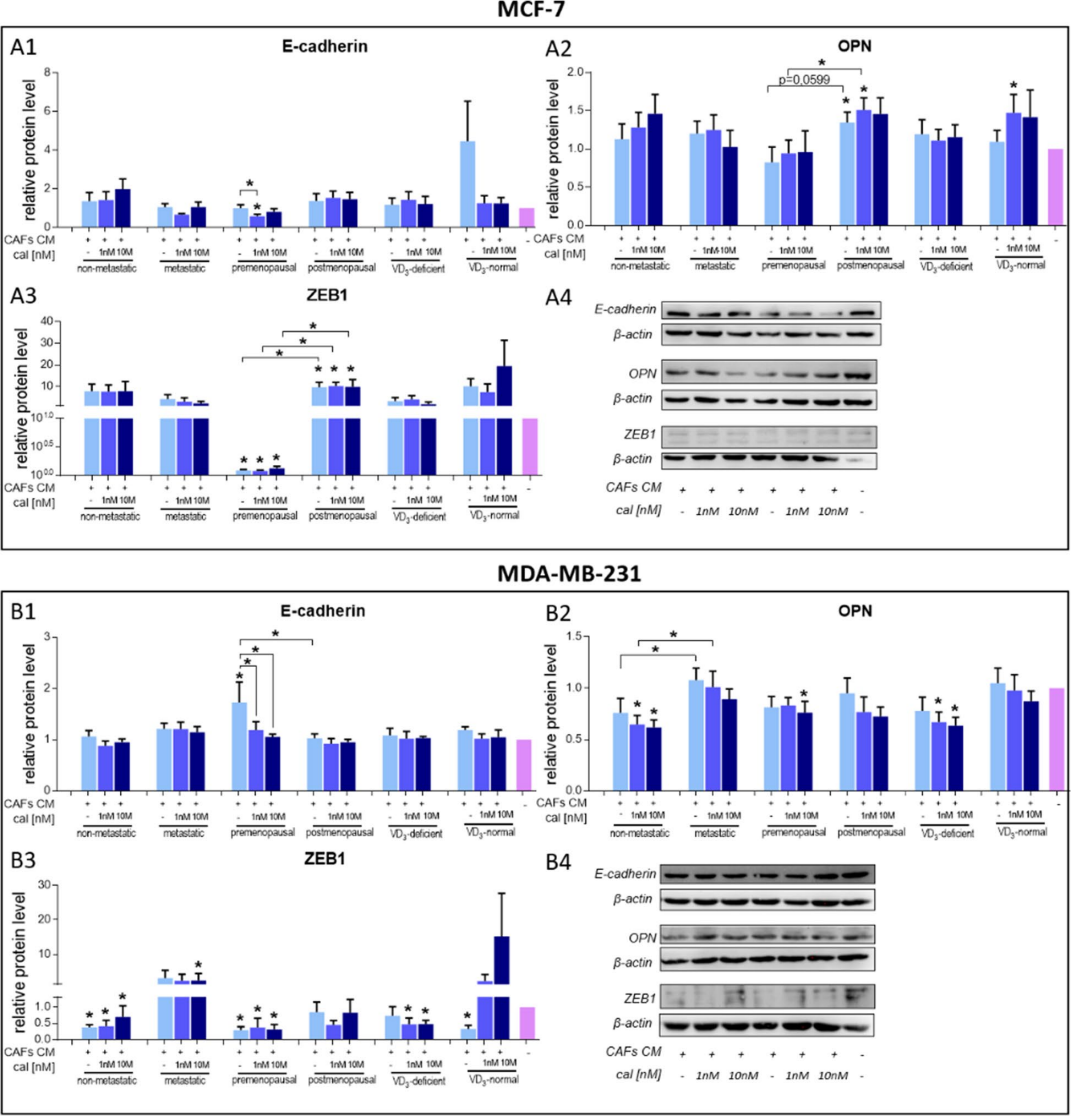
Impact of CAFs on the levels of selected proteins in breast cancer cells. Breast cancer cells: **A** MCF-7 and **B** MDA-MB-231 cells were incubated with conditioned media (CM) from calcitriol-treated CAFs derived from tumors of patients with different clinical characteristics. Levels of the following proteins were assessed: **A1**, **B1** E-cadherin levels, **A2**, **B2** OPN (osteopontin) levels, **A3**, **B3** ZEB1 (zinc finger E-box binding homeobox 1) levels, and A4, **B4** representative cropped blots. Molecular weight of analyzed proteins: E-cadherin—120 kDa (MCF-7) or 40 kDa (intracellular domain, MDA-MB-231), OPN—40 kDa, ZEB1—130 kDa. Full-length blots are presented in Figures S12 and S13 in the Supplementary Materials. CAFs were stimulated with calcitriol (cal) for 72 h prior to CM generation for 24 h. CM was applied to cancer cells for 72 h. Patients were classified into groups according to plasma 25(OH)D_3_ levels (VD3, < 30 ng/mL—deficient (*n* = 10), > 30 ng/mL—normal (*n* = 6)), plasma FSH levels (< 25.8 mIU/mL—premenopausal (*n* = 7), > 25.8 mIU/mL—postmenopausal (*n* = 9)) and regional or distant metastasis presence (if any—metastatic (*n* = 7), otherwise nonmetastatic (h 9)). Densitometric analysis was performed using ImageJ software. The results were normalized to β-actin levels and untreated cancer cells. Data are presented as the mean ± SEM. Statistical analysis was carried out using Student’s *t*-test or the Mann‒Whitney U test for single comparisons and one-way ANOVA or the Kruskal‒Wallis test for multiple comparisons. *p ≤ 0.05 compared to untreated cancer cells

CM from untreated and calcitriol-treated CAFs increased the migration of both breast cancer cell lines (Fig. 9A–B and Figure S10 in Supplementary Materials). Furthermore, CM from 10 nM calcitriol-treated CAFs derived from tumors of postmenopausal or metastasis-free patients decreased the migration of MCF-7 cells (Fig. 9A2). No effect of calcitriol treatment on CAFs was observed in the migration of MDA-MB-231 cells (Fig. 9B2). Besides migration assessment, the protein levels of E-cadherin, OPN, and ZEB1 were measured in MCF-7 and MDA-MB-231 cells after incubation with CAF CM (the results for all CAFs are presented in Figure S11 in the Supplementary Materials). Incubation with CM from CAFs derived from premenopausal and postmenopausal patients induced distinct effects. In both breast cancer cell lines, MCF-7 and MDA-MB-231, CM from calcitriol-treated CAFs from premenopausal women’s tumors decreased the level of E-cadherin, while CM from CAFs from postmenopausal patients did not change the E-cadherin level (Fig. 10A1, A4, B1 and B4). CM from CAFs from tumors of postmenopausal patients increased the levels of OPN and ZEB1 in MCF-7 cells, and CAFs’ calcitriol treatment did not affect these levels (Fig. 10A2–A4). Moreover, OPN and ZEB1 levels were higher in MCF-7 cells treated with CM from CAFs from postmenopausal women than in MCF-7 cells treated with CM from CAFs from premenopausal women (Fig. 10A2–A3). CM from CAFs from premenopausal patients reduced ZEB1 levels in both MCF-7 and MDA-MB-231 cells (Fig. 10A3-A4 and B3-B4). CM from 1 nM calcitriol-treated CAFs from patients with normal levels of vitamin D_3_ increased OPN levels in MCF-7 cells (Fig. 10A2 and A4), while CM from calcitriol-treated CAFs from patients with vitamin D_3_ deficiency decreased OPN and ZEB1 levels in MDA-MB-231 cells (Fig. 10B2–B4). CM from calcitriol-treated CAFs of nonmetastatic patients also reduced OPN levels in MDA-MB-231 cells (Fig. 10B2 and B4). Furthermore, irrespective of calcitriol treatment, CM from CAFs from nonmetastatic patients decreased ZEB1 levels in MDA-MB-231 cells. Moreover, 10 nM calcitriol-treated CAF CM increased ZEB1 levels in these cells (Fig. 10B3-B4).

## Discussion

Women diagnosed with breast cancer usually have low levels of vitamin D_3_, measured by assessing the concentration of its metabolite, 25(OH)D_3_ in plasma [7]. Among the patients who participated in this study, 70% (90/127) had plasma 25(OH)D_3_ levels below 30 ng/ml, which is considered a threshold between normal and deficient plasma levels of this metabolite, indicating vitamin D_3_ deficiency in the human body [31]. While calcitriol is known for its anticancer activity in vitro, some clinical studies have suggested an increased risk of breast cancer in patients with high plasma 25(OH)D_3_ levels exceeding 99 nmol/L (approximately 40 ng/mL) or low levels below 52 nmol/L (approximately 21 ng/mL) [6]. On the other hand, the study by Ganji et al. suggested that plasma 25(OH)D_3_ levels above 99 nmol/L are associated with a reduced risk of breast cancer in postmenopausal women [4]. In addition, our previous study conducted on mouse breast cancer models indicated that high plasma vitamin D_3_ metabolite levels, as well as calcitriol treatment, result in greater metastatic potential of invasive breast cancers (4T1 model) [32]. In this study, we found that normal plasma 25(OH)D_3_ levels were associated with increased levels of TGFβ1 and β-catenin in tumor tissues (protein level assessed in the tumor tissues without sorting of tumor cells) compared to cases of vitamin D_3_ deficiency. TGFβ1 might have antitumoral effects [33], but some studies suggest that both β-catenin and TGFβ1 can play tumor-supporting roles in cancer [34, 35]. Additionally, activation of β-catenin can promote TGFβ-dependent activation of fibroblasts, which may further promote cancer progression [36, 37]. However, in this study, levels of β-catenin and TGFβ1 in tumors did not correlate with the level of CAF infiltration. Tumors in patients with normal plasma 25(OH)D_3_ levels were also characterized by lower levels of CYP24A1 (assessed in the tumor tissues, not on cancer cells), indicating reduced local degradation of calcitriol [38]. Furthermore, in specimens from vitamin D_3_-deficient patients, we observed an inverse association between CAF tumoral infiltration and CYP24A1 levels, while CAF infiltration was positively associated with tumor OPN levels (assessed similarly to β-catenin and TGFβ1). OPN is recognized as a biomarker of tumor progression [39] and can induce the transformation of mesenchymal stem cells (MSCs) or residual fibroblasts into CAFs [40, 41]. These observations may indicate the tumor-supporting role of intratumoral calcitriol in breast cancer, both in vitamin D_3_-deficient and vitamin D_3_-normal patients, which contradicts commonly published in vitro results [2].

To date, only one study has investigated the impact of calcitriol on human breast CAFs [28]. According to Campos et al., the effects of calcitriol on breast CAFs correspond to its influence on breast cancer cells. Calcitriol treatment reduced the procancerous CAF phenotype by downregulating genes involved in proliferation (NRG1, WNT5A, PDGFC) and upregulating genes associated with immune regulation (NFKBIA, TREM-1) [28]. However, these results were observed using a high, physiologically unattainable calcitriol concentration of 100 nM, and similar results were not achieved with 0.5 nM calcitriol [28]. Moreover, our previous study involving mouse breast cancer models indicated that a vitamin D_3_-rich diet and calcitriol treatment of mice on a standard diet supported the development of more invasive CAF phenotypes [27]. In this study, we isolated CAFs from human breast cancer tissues and established primary cultures, which were then ex vivo stimulated with 1 nM (physiologically attainable) or 10 nM (commonly used in in vitro/ex vivo studies) calcitriol [42–44]. Our observations showed that calcitriol reduces CAF viability, with inhibitory effects becoming evident at concentrations starting from 10 nM. Similar findings have been reported by Gorchs et al. [23] and Sherman et al. [20] in CAFs from pancreatic cancer, where 100 nM calcipotriol (a calcitriol analog) decreased the CAF proliferation rate [20, 23]. Campos et al. also noted that 100 nM calcitriol reduced the expression of genes involved in the proliferation of breast CAFs. Moreover, our observations were that only calcitriol concentrations exceeding 10 nM (100 nM and 1000 nM) reduced the viability of CAFs isolated from tumors of premenopausal patients or patients with vitamin D_3_ deficiency. It is possible that the difference in the response to calcitriol antiproliferative activity between CAFs derived from vitamin D_3_-deficient or -normal patients could be associated with a higher degradation of calcitriol in the tumors of vitamin D_3_-deficient patients (indicated by a higher level of CYP24A1).

The results of this study revealed both tumor-supporting and tumor-restraining actions of calcitriol in the context of CAFs derived from different patient groups. Through the secretion of chemokines like CCL2 or CXCL12, CAFs play a crucial role in recruiting monocytes/macrophages and neutrophils into the TME leading to their polarization into protumoral phenotypes such as M2 macrophages, myeloid-derived suppressor cells, or N2 neutrophils. These cells might subsequently reduce T-cell infiltration and the cytotoxic activities of dendritic, NK, or T CD8^+^ cells while promoting T_reg_ cell differentiation and a shift from a T_h1_ to T_h2_ immune response [45–47]. Although, CCL2 production and gene expression decreased in CAFs derived from all patient groups, secretion of CXCL12 was not changed or even increased in CAFs from tumors of vitamin D_3_-deficient patients. In line with the presented results, Ferrer-Mayorga et al. [25] observed a calcitriol-dependent decrease in CCL2 mRNA expression in CAFs of colorectal cancer. Furthermore, TNC^+^ CAFs or PDPN^+^ CAFs can be involved in the recruitment of monocytes or macrophages [48, 49], with TNC impairing T-cell activation, proliferation, and cytokine production [50]. In a previous study, we observed increased TNC and PDPN levels associated with elevated plasma vitamin D_3_ metabolite levels in mice fed a vitamin D_3_-rich diet or administered calcitriol, both in lung fibroblasts from mice bearing 4T1 metastatic cancer cells and in CAFs from E0771 tumors [27, 32]. In the majority of cases, calcitriol decreased TNC production and its gene expression but increased *PDPN* expression. However, in CAFs derived from tumors of postmenopausal patients, 10 nM calcitriol increased TNC secretion and PDPN expression. In CAFs isolated from nonmetastatic tumors, 10 nM calcitriol enhanced PDPN levels. Additionally, the level of IDO1 was decreased in CAFs isolated from tumors of vitamin D_3_-deficient or premenopausal patients. IDO1 activity expressed in CAFs, which leads to tryptophan degradation and kynurenine production, may result in T lymphocyte dysfunction, T_c_ cell apoptosis, or differentiation into T_reg_ cells [51–53]. Furthermore, MMP-2 or MMP-9, by degrading the ECM, can release an active form of TGFβ, which is engaged in restricting the antitumoral immune response of T cells [54]. MMP-9 can also inhibit T-cell proliferation by depleting the IL-2 receptor from the lymphocyte surface [55]. According to Kim et al., calcitriol reduces MMP-9 production in fibroblasts directly and indirectly through the activation of its inhibitors, TIMP1 and TIMP2 [56]. Our study observed a downregulation of MMP-9 mRNA and a decrease in MMP-2 activity in CAFs derived from tumors of all patient groups. However, MMP-9 activity was reduced only in CAFs from postmenopausal patients, and an increase in MMP-9 secretion was observed in CAFs from tumors of nonmetastatic patients. Conversely, the level of TGFβ1 decreased after calcitriol treatment in CAFs from metastatic tumors. Therefore, ex vivo calcitriol treatment resulted in a decreased immunosuppressive phenotype in CAFs derived from breast tumors of vitamin D_3_-deficient (decreased CCL2, TNC, IDO1, MMP-2) and vitamin D_3_-normal (CCL2, TNC, MMP-2), premenopausal (CCL2, TNC, IDO1, MMP-2), and postmenopausal patients (CCL2, TNC—only 1 nM, MMP-2 or MMP-9), as well as in CAFs isolated from metastatic (CCL2, TNC, TGFβ, MMP-2) or nonmetastatic (CCL2, TNC, MMP-2) tumors.

However, some indications of immunosuppression promotion following calcitriol treatment were observed in nonmetastatic patients (increased PDPN expression and MMP-9 secretion) and postmenopausal patients (an increase in PDPN expression). In a study by Gorchs et al., calcipotriol induced immunosuppression in pancreatic cancer TME, which was even more pronounced in the presence of CAFs [23]. Moreover, subcutaneous calcitriol injections in 4T1-bearing mice led to an increased number of monocytes in the bloodstream and a higher ratio of proinflammatory (LyC6^high^CXCR1^low^CCR2^+^) to anti-inflammatory (LyC6^low^CXCR1^high^) spleen monocytes [57]. Calcitriol administration also led to an increased T_h2_ or T_reg_ immune response type in the spleen and decreased CD4^+^ and CD8^+^ T lymphocytes in the plasma and mouse mammary gland tumor tissue [58, 59]. Increased numbers of immunosuppressive cells in the TME (M2 macrophages, N2 neutrophils, MDSCs, T_reg_ cells), a T_h1_ to T_h2_ shift, inhibition of cytotoxic cell activities (CD8^+^ T cells, NK cells), or apoptosis of lymphocytes enable cancer cells to evade an antitumor immune response. The immunosuppressive TME is actively engaged in angiogenesis, invasion, or premetastatic niche formation [60]. Together with *PDPN* and *TIMP1* expression, *SPP1* mRNA (encoding OPN) increased after calcitriol treatment of CAFs isolated from tumors of all patient groups. Some previous studies reported that in vitro calcitriol treatment enhances the secretion of OPN in BALB/3T3 fibroblasts [57]. Campos et al. reported that *SPP1* mRNA is commonly expressed in normal breast fibroblasts and CAFs and is upregulated after calcitriol treatment [28]. However, the upregulation of *SSP1* mRNA in CAFs was not accompanied by an increase in OPN protein level.

Similar to findings in studies by Ferrer-Mayorga et al. on colon CAFs and Kim et al. on lung fibroblasts, our research revealed a calcitriol-dependent increase in one of the tissue metalloproteinases, TIMP1 (TIMP3 in [25, 26] or TIMP1 and TIMP2 in [56]). This effect was independent of the origin of CAFs. In general, TIMPs inhibit MMPs, so the increase in their level could be attributed to fighting against tumor progression [61]. Nonetheless, increased TIMP expression also correlates with poor prognosis in patients diagnosed with TNBC [62]. The TIMP1/CD63/β1-integrin complex activates MAPK, FAK-PI3K, or YAP/TAZ signaling, which promotes cancer cell proliferation, growth, survival, migration, and EMT, regulates differentiation and inhibits apoptosis of cancer cells, ultimately leading to tumor progression and metastasis [63–70]. Conversely, the reduction in CCL2, MMP-2, and TNC production/activity induced by calcitriol in CAFs of almost every origin is associated with impairment of the same processes that promote metastasis, such as CCL2-driven macrophage recruitment involved in angiogenesis and intravasation of cancer cells [71, 72]. Reduced interaction between CCL2 and CCR2 on breast cancer cells can hinder their migration and survival, possibly through diminished activation of Smad3 or MAPK signaling [73]. Decreased TNC production can induce cancer cell apoptosis [74]. The same impact of calcitriol was observed in epithelial and breast cancer cells [75]. A reduction in MMP-2 or TNC may result in inhibition of the proliferation, migration, and invasion of cancer cells as well as decreased angiogenesis and premetastatic niche formation [76, 77]. In cases of CAFs isolated from tumors of patients with normal plasma 25(OH)D_3_ levels, calcitriol exhibited predominantly antitumoral effects, including decreased CCL2, TNC, and OPN production (but increased *SPP1* mRNA) and MMP-2 activity but increased TIMP1 levels. However, we observed that the effect on CAF proliferation inhibition was achieved only at high calcitriol concentrations. On the other hand, CAFs from vitamin D_3_-deficient patients, which are more sensitive to calcitriol’s antiproliferative action apart from decreased CCL2, TNC, and MMP-2 and increased TIMP-1, are also characterized by decreased IDO1 expression. The effect of calcitriol also varied between CAFs derived from tumors of patients with different menopausal statuses or metastases. In CAFs from premenopausal patients, calcitriol reduced CAF protumoral activities through decreases in CCL2, TNC, and *MMP9* mRNA (no effect on protein levels) levels and MMP-2 production and promoted CAF protumoral properties by increasing TIMP1 levels or upregulating *SPP1* and *PDPN* mRNAs (no effect on protein levels). In CAFs from tumors of postmenopausal women, calcitriol increased PDPN, TIMP1, and TNC levels and upregulated *SPP1* mRNA (no effect on protein level). However, the calcitriol effect on TNC was observed only after treatment with 10 nM calcitriol. Calcitriol also decreased CCL2, MMP-2, MMP-9, and TNC (1 nM). Interestingly, the effect of calcitriol treatment was more favorable in the case of CAFs isolated from metastatic tumors than CAFs from nonmetastatic tumors. In CAFs from metastatic tumors, a decrease in CCL2, MMP-2, TNC, OPN, and TGFβ levels and an increase in only TIMP1, *PDPN*, and *SPP1* mRNA levels (without an effect on protein levels) was observed after calcitriol treatment. In CAFs from nonmetastatic tumors, increased levels of PDPN, TIMP1, MMP-9, *PDPN,* and *SPP1* mRNAs were observed together with decreases in CCL2 and TNC levels and MMP-2 activity. It is difficult to assess the relative influence of specific cytokines on the processes leading to breast tumor growth, progression, or metastasis. Without studies that closely examine the mechanisms underlying calcitriol’s effect and that also consider other variables affecting tumor development, migration, invasion, EMT, and angiogenesis, it is not possible to unambiguously determine the role of calcitriol in breast CAFs.

Breast cancer cells are sensitive to calcitriol in vitro. Breast cancer cells treated with calcitriol decrease the expression of ZEB1, N-cadherin, vimentin, or integrins and increase the level of E-cadherin, suggesting that calcitriol reduces the EMT process [78]. Sherman et al. observed that calcipotriol treatment inhibits the protumoral activity of CAFs by reducing CAF-induced expression of genes involved in proliferation, survival, EMT, or chemoresistance (*CXCL1*, *CCND1*, *CDK1*, *SHH*, *BIRC5*, and *AURKB*) in pancreatic cancer cells (MIAPCa-2) [20]. Based on these observations, we decided to determine how CAFs derived from the tumors of patients with different clinical characteristics influence human breast cancer cells and whether calcitriol changes these cells. The distinct effect of calcitriol on the protumoral activities of CAFs was observed when two breast cancer cell lines, representing different molecular subtypes, were incubated with CM from calcitriol-treated CAFs. CAFs promote the migration of both breast cancer cell lines and modulate the concentration of proteins important for tumor progression in these cells. In MCF-7 cells, representing luminal A breast cancers, the promigratory capacities of CAFs derived from tumors of postmenopausal and nonmetastatic patients were attenuated by 10 nM calcitriol. This effect was observed although these two groups of CAFs possessed increased surface expression of PDPN, which is recognized as a cancer cell migration-promoting factor in CAFs from lung, pancreatic, or some breast tumors [34, 79–81]. According to Niemiec et al., PDPN^+^ CAFs isolated from HER2-overexpressing breast carcinomas facilitate the migration of breast tumor cells [81], but Suchanski et al. described that PDPN expressed on the surface of human fibroblastic cell lines (MSU1.1 and Hs 578Bst) does not change the migration rate of cells that were also used in these studies—MCF-7 and MDA-MB-23 [82]. In our studies, calcitriol stimulation did not change the effects of CAFs on MDA-MB-231 TNBC cells. However, calcitriol treatment of CAFs from tumors of premenopausal, vitamin D_3_-deficient, or nonmetastatic patients resulted in decreased OPN levels in MDA-MB-231 cells incubated with CM from these CAFs. Simultaneously, CM from calcitriol-treated CAFs from tumors of vitamin D_3_-deficient patients reduced ZEB1 levels in MDA-MB-231 cells. The inhibition of CAF-induced expression of OPN or ZEB1 in breast cancer cells confirms the antitumoral activities of calcitriol in CAFs because OPN and ZEB1 are involved in various processes leading to breast tumor progression [83–85]. Unfortunately, these observations are the only ones indicating antitumoral calcitriol activity in the context of its impact on CAFs. The CM from calcitriol-treated CAFs derived from premenopausal patients reduced E-cadherin levels in MCF-7 (1 nM) and MDA-MB-231 (1 nM and 10 nM) cells. E-cadherin is a marker of the epithelial phenotype, and its decreased level suggests EMT induction [86]. Moreover, increased OPN levels were observed in MCF-7 cells incubated with CM from 1 nM calcitriol-treated CAFs from tumors of patients with normal vitamin D_3_ plasma levels. Interestingly, independent of calcitriol treatment, CAFs from tumors of premenopausal patients decreased ZEB1 levels in MCF-7 and MDA-MB-231 cells. Analogously, MDA-MB-231 cell incubation with CM from CAFs derived from nonmetastatic tumors resulted in ZEB1 reduction. On the other hand, CM from CAFs isolated from tumors of postmenopausal patients led to an increase in ZEB1 in MCF-7 cells. However, Matsumura et al. found that fibroblasts could promote the generation of clusters of diverse cancer cell populations, with an epithelial phenotype (E^hi^) characterized by high E-cadherin levels and low ZEB1 levels and a mixed epithelial-mesenchymal phenotype (E/M) characterized by low E-cadherin levels and high ZEB1 levels [87]. Due to partially maintained E-cadherin expression, cancer cells with the E/M phenotype could bind to E^hi^ cancer cells (with high adhesion capacities), and together, these two cancer cell populations are conducive to invasion and metastasis [87].

## Conclusion

The results of calcitriol treatment in CAFs derived from tumors of patients with different clinical characteristics did not exhibit a straightforward, unidirectional effect. While it led to a reduction in the levels of certain proteins involved in the promotion of proliferation, migration, EMT, tumor angiogenesis, or metastasis, a simultaneous increase was noted in the levels of other proteins with overlapping functionalities. This complexity could be attributed to the inherent heterogeneity within CAF populations, suggesting that calcitriol’s effects might vary across CAF subtypes. Here, in the case of CAFs derived from vitamin D3-deficient patients, premenopausal individuals, and patients with or without metastases, predominant antitumoral calcitriol effects were observed. This anticancer activity of calcitriol was reflected by the modulation of CAFs’ impact on metastases or immune escape of cancer cells. Moreover, the impact of calcitriol not only depends on CAF characteristics but is also determined by the specific type of cancer cells with which CAFs interact. To comprehensively understand the impact of calcitriol on breast CAFs, further experiments must be performed.

### Supplementary Information

**Additional file 1: Table S1.** Selected clinical characteristics of the patients involved in the study. **Table S2.** Algorithm used in this study for scoring CAFs infiltration. **Table S3.** List of genes and corresponding probes used in screening PCR array cards. **Table S4.** CAFs statuses in tumor tissues from patients with different clinical characteristics. **Figure S1.** VDR, CYP27B1, CYP24A1 levels in tumor tissues from patients with different clinical characteristics. **Figure S2.** Uncropped blot images corresponding to cropped blots presented in Figure 1 in the manuscript: OPN, TGFβ and β-catenin levels in tumor tissues from patients with different clinical characteristics. **Figure S3.** Uncropped blot images corresponding to cropped blots presented in Figure S1 in the Supplementary Materials: VDR, CYP27B1, CYP24A1 levels in tumor tissues from patients with different clinical characteristics. **Figure S4.** Gating strategy for CAFs phenotype assessment using flow cytometry. **Figure S5.** Characterization of CAFs phenotype. **Figure S6.** Impact of calcitriol on the phenotype of CAFs derived from tumors of patients with different clinical characteristics. **Figure S7.** The expression matrix of 61 genes from 19 selected CAFs cultures. **Figure S8.** Uncropped blot images corresponding to cropped blots presented in Figure 7 in the manuscript: Selected protein levels in calcitriol-treated CAFs derived from tumors of patients with different clinical characteristics. **Figure S9.** Uncropped gel images corresponding to cropped gels presented in Figure 8 in the manuscript: Gelatinase activity in calcitriol-treated CAFs derived from tumors of patients with different clinical characteristics. **Figure S10.** Representative photos of the migration of breast cancer cells incubated with conditioned media (CM) from calcitriol-treated CAFs. **Figure S11.** Impact of CAFs on the levels of selected proteins in breast cancer cells. **Figure S12.** Uncropped blot images corresponding to the cropped blots presented in Figure 10 in the manuscript: CAF impact on the levels of selected proteins in breast cancer cells: MCF-7. **Figure S13.** Uncropped blot images corresponding to the cropped blots presented in Figure 10 in the manuscript: CAF impact on the levels of selected proteins in breast cancer cells: MDA-MB-231.

## Data Availability

All data supporting the results are available in the manuscript and supplementary file.

## Abbreviations

αSMA: Alpha-smooth muscle actin
CAFs: Cancer-associated fibroblasts
CCL2: C–C motif chemokine ligand 2
CXCL12: C-X-C motif chemokine ligand 12
CYP24A1: Cytochrome P450 family 24 subfamily A member 1
CYP27B1: Cytochrome P450 family 27 subfamily B member 1
ECM: Extracellular matrix
FSH: Follicle-stimulating hormone
FSP1: Fibroblast specific protein 1
IDO1: Idoleamine 1
MMP: Metalloproteinase
OPN: Osteopontin
PDPN: Podoplanin
SPP1: Secreted phosphoprotein
TGFβ1: Transforming growth factor β 1
TIMP1: Tissue metalloproteinase inhibitor 1
TME: Tumor microenvironment
TNC: Tenascin C
VDR: Vitamin D receptor
