# Supplementary material for: Dual effect of vitamin D3 on breast cancer-associated fibroblasts
Source: BMC Cancer. 2024 Feb 15;24:209. doi: 10.1186/s12885-024-11961-z (PMC10868064; doi:10.1186/s12885-024-11961-z)
Supplement: Supplementary file 1 — Additional file 1: Table S1. Selected clinical characteristics of the patients involved in the study. Table S2. Algorithm used in this study for scoring CAFs infiltration. Table S3. List of genes and corresponding probes used in screening PCR array cards. Table S4. CAFs statuses in tumor tissues from patients with different clinical characteristics. Figure S1. VDR, CYP27B1, CYP24A1 levels in tumor tissues from patients with different clinical characteristics. Figure S2. Uncropped blot images corresponding to cropped blots presented in Figure 1 in the manuscript: OPN, TGFβ and β-catenin levels in tumor tissues from patients with different clinical characteristics. Figure S3. Uncropped blot images corresponding to cropped blots presented in Figure S1 in the Supplementary Materials: VDR, CYP27B1, CYP24A1 levels in tumor tissues from patients with different clinical characteristics. Figure S4. Gating strategy for CAFs phenotype assessment using flow cytometry. Figure S5. Characterization of CAFs phenotype. Figure S6. Impact of calcitriol on the phenotype of CAFs derived from tumors of patients with different clinical characteristics. Figure S7. The expression matrix of 61 genes from 19 selected CAFs cultures. Figure S8. Uncropped blot images corresponding to cropped blots presented in Figure 7 in the manuscript: Selected protein levels in calcitriol-treated CAFs derived from tumors of patients with different clinical characteristics. Figure S9. Uncropped gel images corresponding to cropped gels presented in Figure 8 in the manuscript: Gelatinase activity in calcitriol-treated CAFs derived from tumors of patients with different clinical characteristics. Figure S10. Representative photos of the migration of breast cancer cells incubated with conditioned media (CM) from calcitriol-treated CAFs. Figure S11. Impact of CAFs on the levels of selected proteins in breast cancer cells. Figure S12. Uncropped blot images corresponding to the cropped blots presented in Figure 10 in [file 12885_2024_11961_MOESM1_ESM.docx]

# Supplementary Materials

DUAL EFFECT OF VITAMIN D_3_ ON BREAST CANCER ASSOCIATED FIBROBLASTS

Natalia Łabędź^1,2^, Artur Anisiewicz^1^, Martyna Stachowicz-Suhs^1^, Joanna Banach^1^, Dagmara Kłopotowska^1^, Adam Maciejczyk^3,4^, Patrycja Gazińska^2,5^, Aleksandra Piotrowska^6^, Piotr Dzięgiel^6^, Rafał Matkowski^3,4^, Joanna Wietrzyk^1^

^1^ Department of Experimental Oncology, Hirszfeld Institute of Immunology and Experimental Therapy, Weigla 12, 53-114 Wroclaw, Poland: [natalia.labedz@hirszfeld.pl](mailto:natalia.labedz@hirszfeld.pl), [a.anisiewicz@captortherapeutics.com](mailto:a.anisiewicz@captortherapeutics.com), [martyna.stachowicz@hirszfeld.pl](mailto:martyna.stachowicz@hirszfeld.pl), [joanna.banach@hirszfeld.pl](mailto:joanna.banach@hirszfeld.pl), [dagmara.klopotowska@hirszfeld.pl](mailto:dagmara.klopotowska@hirszfeld.pl), [joanna.wietrzyk@hirszfeld.pl](mailto:joanna.wietrzyk@hirszfeld.pl)

^2^ Łukasiewicz Research Network—PORT Polish Center for Technology Development, Stabłowicka 147, 54-066 Wrocław, Poland: [natalia.labedz@port.lukasiewicz.gov.pl](mailto:natalia.labedz@port.lukasiewicz.gov.pl), [patrycja.gazinska@port.lukasiewicz.gov.pl](mailto:patrycja.gazinska@port.lukasiewicz.gov.pl)

^3^ Department of Oncology, Wroclaw Medical University, Pl. Ludwika Hirszfelda 12, 53-413 Wroclaw, Poland: [rafal.matkowski@umw.edu.pl](mailto:rafal.matkowski@umw.edu.pl), [adam.maciejczyk@umw.edu.pl](mailto:adam.maciejczyk@umw.edu.pl)

^4^ Lower Silesian Oncology, Pulmonology and Hematology Center, Pl. Ludwika Hirszfelda 12,53-413 Wroclaw, Poland: [matkowski.rafal@dco.com.pl](mailto:matkowski.rafal@dco.com.pl), [adam.maciejczyk@dco.com.pl](mailto:adam.maciejczyk@dco.com.pl)

^5^ Research Oncology, Division of Cancer Studies, King's College London, Great Maze Pond, London SE1 3SS, United Kingdom

^6^ Division of Histology and Embryology, Department of Human Morphology and Embryology, Wroclaw Medical University, ul. Chałubińskiego 6a, 50-368 Wroclaw, Poland: [aleksandra.piotrowska@umw.edu.pl](mailto:aleksandra.piotrowska@umw.edu.pl), [piotr.dziegiel@umw.edu.pl](mailto:piotr.dziegiel@umw.edu.pl)

Corresponding author: [natalia.labedz@hirszfeld.pl](mailto:natalia.labedz@hirszfeld.pl)

Table S 1. **Selected clinical characteristics of the patients involved in the study**.

| **No. of patient** | **ER-positive** | **HER 2-positive** | **TNBC** | **Tumor grade (G)** | **25(OH)D_3_ [ng/ml]** | **Vitamin D_3_ level description** | **FSH [mIU/ml]** | **Menopausal status description** |
| --- | --- | --- | --- | --- | --- | --- | --- | --- |
| **1** | Y | N | N | 2 | 39.60 | normal | 82,30 | premenopausal |
| **2** | Y | N | N | 2 | 49.80 | normal | 103.00 | postmenopausal |
| **3** | Y | N | N | 2 | 26.90 | deficient | 3.30 | premenopausal |
| **4** | Y | N | N | 1 | 13.00 | deficient | 37.40 | postmenopausal |
| **5** | Y | N | N | 2 | 21.50 | deficient | 73.40 | postmenopausal |
| **6** | Y | N | N | 2 | 23.10 | deficient | 43.70 | premenopausal |
| **7** | Y | N | N | 2 | 7.14 | deficient | 71.60 | postmenopausal |
| **8** | Y | N | N | 2 | 18.40 | deficient | 54.80 | postmenopausal |
| **9** | Y | N/A | N | 3 | 31.30 | normal | 6.00 | premenopausal |
| **10** | Y | N | N | 2 | 34.10 | normal | 5.80 | premenopausal |
| **11** | Y | N | N | N/A | 25.00 | deficient | 7.10 | premenopausal |
| **12** | Y | N | N | 2 | 19.50 | deficient | 3.20 | premenopausal |
| **13** | Y | N | N | N/A | 15.50 | deficient | 47.80 | postmenopausal |
| **14** | Y | N | N | 2 | 18.60 | deficient | 78.30 | postmenopausal |
| **15** | Y | N | N | 1 | 15.60 | deficient | 63.70 | postmenopausal |
| **16** | Y | N | N | 1 | 45.00 | normal | 4.20 | premenopausal |
| **17** | Y | Y | N | 3 | 23.20 | deficient | 13.00 | premenopausal |
| **18** | Y | N | N | 2 | 18.40 | deficient | 77.60 | postmenopausal |
| **19** | Y | N | N | 2 | 35.40 | normal | 55.10 | postmenopausal |
| **20** | Y | N | N | 3 | 14.70 | deficient | 77.70 | postmenopausal |
| **21** | Y | Y | N | 2 | 38.20 | normal | 61.60 | postmenopausal |
| **22** | Y | N | N | 1 | 23.40 | deficient | 30.30 | postmenopausal |
| **23** | N/A | N/A | N | N/A | 20.70 | deficient | 6.90 | premenopausal |
| **24** | Y | Y | N | 1 | 16.30 | deficient | 20.20 | premenopausal |
| **25** | Y | N | N | 3 | 10.00 | deficient | 60.00 | postmenopausal |
| **26** | Y | N | N | 2 | 20.70 | deficient | 6.80 | premenopausal |
| **27** | Y | N | N | 2 | 20.60 | deficient | 93.10 | postmenopausal |
| **28** | Y | N | N | 3 | 31.70 | normal | 25.50 | postmenopausal |
| **29** | N | N | Y | 3 | 15.60 | deficient | 72.00 | postmenopausal |
| **30** | Y | N | N | 2 | 40.10 | normal | 6.90 | premenopausal |
| **31** | Y | N | N | 2 | 31.50 | normal | 154.00 | postmenopausal |
| **32** | Y | Y | N | 2 | 25.00 | deficient | 32.10 | premenopausal |
| **33** | Y | N | N | 2 | 21.90 | deficient | 6.90 | premenopausal |
| **34** | Y | N | N | 1 | 55.70 | normal | 65.20 | postmenopausal |
| **35** | Y | N | N | 2 | 21.50 | deficient | 90.50 | postmenopausal |
| **36** | Y | N | N | 2 | 28.30 | deficient | 9.50 | premenopausal |
| **37** | Y | Y | N | 2 | 31.70 | normal | 43.40 | postmenopausal |
| **38** | Y | N | N | 3 | 10.00 | deficient | 65.00 | postmenopausal |
| **39** | N/A | N | Y | 3 | 19.60 | deficient | 38.90 | premenopausal |
| **40** | Y | N | N | 1 | 7.88 | deficient | N/A | - |
| **41** | Y | N | N | 2 | 39.00 | normal | 2.50 | premenopausal |
| **42** | Y | N | N | 2 | 13.90 | deficient | 97.10 | postmenopausal |
| **43** | Y | Y | N | 2 | 25.40 | deficient | 60.20 | postmenopausal |
| **44** | Y | N | N | 2 | 17.50 | deficient | 67.20 | postmenopausal |
| **45** | N | Y | N | 3 | 16.80 | deficient | 80.40 | postmenopausal |
| **46** | Y | N | N | 3 | 21.00 | deficient | 78.00 | postmenopausal |
| **47** | Y | N | N | 2 | 11.50 | deficient | 42.20 | premenopausal |
| **48** | Y | Y | N | 3 | 21.20 | deficient | 3.00 | premenopausal |
| **49** | Y | N | N | 1 | 12.70 | deficient | 55.60 | postmenopausal |
| **50** | Y | N | N | 2 | 19.30 | deficient | 13.90 | premenopausal |
| **51** | Y | N | N | 2 | 40.50 | normal | 101.00 | postmenopausal |
| **52** | Y | N | N | 1 | 16.00 | deficient | 72.30 | postmenopausal |
| **53** | Y | Y | N | 3 | 22.20 | deficient | 21.50 | postmenopausal |
| **54** | Y | N | N | 2 | 17.90 | deficient | 36.40 | premenopausal |
| **55** | Y | N | N | 3 | 17.00 | deficient | 53.50 | postmenopausal |
| **56** | Y | N | N | 1 | 24.50 | deficient | 58.10 | postmenopausal |
| **57** | Y | Y | N | 1 | 27.90 | deficient | 105.00 | postmenopausal |
| **58** | Y | N | N | 2 | 36.20 | normal | 57.90 | postmenopausal |
| **59** | Y | N | N | 2 | 12.70 | deficient | 55.90 | postmenopausal |
| **60** | Y | N | N | 2 | 18.40 | deficient | 78.30 | postmenopausal |
| **61** | Y | N | N | 2 | 28.40 | deficient | 58.40 | postmenopausal |
| **62** | Y | N | N | 2 | 10.50 | deficient | 51.90 | postmenopausal |
| **63** | Y | N | N | 2 | 57.70 | normal | 96.90 | postmenopausal |
| **64** | Y | N | N | 1 | 31.50 | normal | 3.80 | premenopausal |
| **65** | Y | N | N | 2 | 33.70 | normal | 24.10 | premenopausal |
| **66** | Y | N | N | 2 | 21.90 | deficient | 6.20 | premenopausal |
| **67** | Y | N | N | 2 | 28.40 | deficient | 44.80 | postmenopausal |
| **68** | Y | N | N | 3 | 12.40 | deficient | 34.50 | postmenopausal |
| **69** | N | N | Y | 2 | 21.00 | deficient | 2.40 | premenopausal |
| **70** | Y | N | N | 2 | 5.60 | deficient | 75.70 | postmenopausal |
| **71** | N | N | N | 3 | 33.10 | normal | 70.20 | postmenopausal |
| **72** | Y | N | N | 2 | 38.90 | normal | 75.60 | postmenopausal |
| **73** | N | N | Y | 2 | 29.10 | deficient | 34.20 | postmenopausal |
| **74** | Y | Y | N | 2 | 9.20 | deficient | 123.00 | postmenopausal |
| **75** | Y | N | N | 2 | 28.80 | deficient | 80.00 | postmenopausal |
| **76** | Y | N | N | 2 | 28.00 | deficient | 63.60 | postmenopausal |
| **77** | Y | N | N | 2 | 2.00 | deficient | 68.9 | postmenopausal |
| **79** | Y | N | N | 1 | 42.70 | normal | 23.30 | premenopausal |
| **80** | Y | N | N | 3 | 15.90 | deficient | 41.20 | postmenopausal |
| **81** | Y | N | N | 2 | 26.00 | deficient | 80.10 | postmenopausal |
| **82** | Y | N | N | 2 | 19.10 | deficient | 73.30 | postmenopausal |
| **83** | Y | Y | N | 2 | 42.80 | normal | 9.90 | postmenopausal |
| **84** | Y | N | N | 2 | 14.90 | deficient | 5.40 | premenopausal |
| **85** | Y | N/A | N | 2 | 8.73 | deficient | 138.00 | postmenopausal |
| **86** | Y | N | N | 2 | 36.80 | normal | 54.60 | postmenopausal |
| **87** | Y | Y | N | 2 | 33.90 | normal | 4.00 | postmenopausal |
| **88** | Y | Y | N | 2 | 41,50 | normal | 7,70 | premenopausal |
| **89** | Y | N | N | 2 | 34.30 | normal | 85.70 | postmenopausal |
| **90** | Y | N | N | 2 | 16.20 | deficient | 62.70 | postmenopausal |
| **91** | N | Y | N | 3 | 41.00 | normal | 71.60 | postmenopausal |
| **92** | Y | N | N | 1 | 50.40 | normal | 5.20 | premenopausal |
| **93** | Y | N | N | 1 | 23.60 | deficient | 9.50 | premenopausal |
| **94** | Y | N | N | 2 | 23.50 | deficient | 12.90 | premenopausal |
| **95** | N/A | Y | N | 2 | 60.10 | normal | 43.20 | postmenopausal |
| **96** | Y | N | N | N/A | 26.10 | deficient | 94.70 | postmenopausal |
| **97** | N | N | Y | 3 | 47.00 | normal | 4.90 | premenopausal |
| **98** | Y | N | N | 2 | 19.20 | deficient | 23.80 | postmenopausal |
| **99** | Y | N | N | 2 | 20.70 | deficient | 67.20 | postmenopausal |
| **100** | Y | N | N | 1 | 67.70 | normal | 82.00 | postmenopausal |
| **101** | Y | Y | N | 3 | 49.30 | normal | 80.30 | postmenopausal |
| **102** | Y | N | N | 1 | 53.90 | normal | 138.00 | postmenopausal |
| **103** | Y | N | N | 1 | 25.60 | deficient | 77.80 | postmenopausal |
| **104** | Y | N | N | 2 | 20.20 | deficient | 52.20 | postmenopausal |
| **105** | Y | N | N | 2 | 10.60 | deficient | 22.80 | postmenopausal |
| **106** | Y | N | N | 2 | 13.90 | deficient | 69.40 | postmenopausal |
| **107** | N | N | Y | 2 | 20.40 | deficient | 112.00 | postmenopausal |
| **108** | N/A | Y | N | 3 | 26.10 | deficient | 63.90 | postmenopausal |
| **109** | Y | N | N | 2 | 22.70 | deficient | 56.90 | postmenopausal |
| **110** | Y | N | N | 2 | 9.81 | deficient | 76.00 | postmenopausal |
| **111** | Y | N | N | 2 | 29.10 | deficient | 70.90 | postmenopausal |
| **112** | Y | Y | N | 2 | 23.70 | deficient | 6.40 | premenopausal |
| **113** | N | Y | N | 3 | 24.30 | deficient | 88.20 | postmenopausal |
| **114** | Y | N/A | N | 2 | 23.70 | deficient | 7.00 | premenopausal |
| **115** | Y | Y | N | 2 | 32.20 | normal | 33.90 | postmenopausal |
| **116** | Y | Y | N | 2 | 9.30 | deficient | 69.60 | postmenopausal |
| **117** | Y | N | N | 3 | 42.10 | normal | 89.70 | postmenopausal |
| **118** | Y | N | N | 2 | 16.30 | deficient | 79.40 | postmenopausal |
| **119** | Y | N | N | 2 | 31.50 | normal | 68.50 | postmenopausal |
| **120** | Y | N | N | 1 | 20.70 | deficient | 97.60 | postmenopausal |
| **121** | Y | Y | N | 2 | 14.10 | deficient | 105.00 | postmenopausal |
| **122** | Y | N/A | N | 2 | 32.60 | normal | 8.40 | premenopausal |
| **123** | Y | N | N | 2 | 29.20 | deficient | 65.90 | postmenopausal |
| **124** | Y | N | N | 2 | 9.49 | deficient | 89.40 | postmenopausal |
| **125** | Y | N | N | 2 | 43.10 | normal | 73.00 | postmenopausal |
| **126** | Y | Y | N | 3 | 21.70 | deficient | 3.90 | premenopausal |
| **127** | Y | N | N | 2 | 14.90 | deficient | 85.10 | postmenopausal |
| **128** | Y | Y | N | 2 | 24.40 | deficient | 6.40 | premenopausal |

ER – Estrogen receptor, HER2 - human epidermal growth factor receptor 2, TNBC – triple-negative breast cancer.

Table S 2. **CAFs infiltration scoring algorithm used in this study**.

| No. | CAFs infiltration scoring algorithm |
| --- | --- |
| 1. | **IF** the sum of activation and density levels does not exceed 3 -> 1 (low), |
| 2. | **BUT** specimens with low fibroblasts activation level (labeled as “1”) -> max 2 (medium-low), |
| 3. | **IF** the sum of activation and density levels is 6 -> 3 (medium), |
| 4. | **BUT** specimens with medium-low fibroblasts activation (2) -> max 3, |
| 5. | **IF** the sum of activation and density levels is 7 or 8 -> 4 (medium-high), |
| 6. | **IF** the sum of activation and density levels is 9 or 10 -> 5 (high), |
| 7. | **BUT** specimens with high fibroblasts activation level (5) -> minimum 4 (medium-high). |

Table S 3. **List of genes and corresponding probes used in screening PCR array cards.**

|  | **Gene** | **Probe** |  | **Gene** | **Probe** |
| --- | --- | --- | --- | --- | --- |
| **endogenous control** | *ACTB* | Hs99999903_m1 | **analyzed genes** | *MMP11* | Hs00171829_m1 |
|  | *GAPDH* | Hs99999905_m1 |  | *MMP1* | Hs00899658_m1 |
|  | *RPLP0* | Hs99999902_m1 |  | *MMP2* | Hs00234422_m1 |
|  | *SDHA* | Hs00188166_m1 |  | *MMP3* | Hs00968308_m1 |
| **analyzed genes** | *ACTA2* | Hs05005341_m1 |  | *MMP9* | Hs00234579_m1 |
|  | *CAV1* | Hs00971716_m1 |  | *NFKB1* | Hs00765730_m1 |
|  | *CCL2* | Hs00234140_m1 |  | *PDGFA* | Hs00234994_m1 |
|  | *CDH1* | Hs00170423_m1 |  | *PDGFB* | Hs00234042_m1 |
|  | *CDKN1A* | Hs00355782_m1 |  | *PDPN* | Hs00366766_m1 |
|  | *CDKN2A* | Hs00923894_m1 |  | *PECAM1* | Hs00169777_m1 |
|  | *CSF1* | Hs00174164_m1 |  | *PLAU* | Hs00170182_m1 |
|  | *CTGF* | Hs00170014_m1 |  | *PTEN* | Hs02621230_s1 |
|  | *CXCL12* | Hs00171022_m1 |  | *PTGES2* | Hs00228159_m1 |
|  | *CXCL1* | Hs00236937_m1 |  | *PTGS2* | Hs00153133_m1 |
|  | *CXCL2* | Hs00601975_m1 |  | *RARA* | Hs00940446_m1 |
|  | *CXCL8* | Hs00174103_m1 |  | *RXRA* | Hs01067640_m1 |
|  | *CYP24A1* | Hs00167999_m1 |  | *S100A4* | Hs00243202_m1 |
|  | *CYP27B1* | Hs00168017_m1 |  | *SHH* | Hs00179843_m1 |
|  | *CYR61* | Hs00155479_m1 |  | *SPARC* | Hs00277762_m1 |
|  | *DES* | Hs00157258_m1 |  | *SPP1* | Hs00959010_m1 |
|  | *FAP* | Hs00990806_m1 |  | *TGFB1* | Hs00998133_m1 |
|  | *FGF2* | Hs00960934_m1 |  | *TGFBR2* | Hs00234253_m1 |
|  | *FGF7* | Hs00940253_m1 |  | *THBS1* | Hs00962914_m1 |
|  | *FN1* | Hs00415008_m1 |  | *THEM4* | Hs00940012_g1 |
|  | *HAS2* | Hs00193435_m1 |  | *THY1* | Hs00174816_m1 |
|  | *HGF* | Hs00900066_m1 |  | *TIMP1* | Hs00171558_m1 |
|  | *IDO1* | Hs00158032_m1 |  | *TNC* | Hs01115664_m1 |
|  | *IGF2* | Hs04188276_m1 |  | *TP53* | Hs00153349_m1 |
|  | *IL1B* | Hs01555413_m1 |  | *TSLP* | Hs00263639_m1 |
|  | *IL6* | Hs99999032_m1 |  | *VDR* | Hs01045840_m1 |
|  | *LAMA1* | Hs01074480_m1 |  | *VEGFA* | Hs00900055_m1 |

ACTB – β-actin, GAPDH - glyceraldehyde 3-phosphate dehydrogenase, RPLP0 - ribosomal protein lateral stalk subunit P0, SDHA - succinate dehydrogenase complex flavoprotein subunit A, ACTA2 - α-actin 2, CAV1 - caveolin 1, CCL2 - C-C motif chemokine ligand 2, CDH1 - cadherin 1, CDKN1A - cyclin dependent kinase inhibitor 1A, CDKN2A – cyclin dependent kinase inhibitor 2A, CSF1 – colony stimulating factor 1, CTGF – connective tissue growth factor, CXCL12 – C-X-C motif chemokine ligand 12, CXCL1 – C-X-C motif chemokine ligand 1, CXCL2 - C-X-C motif chemokine ligand 2, CXCL8 – C-X-C motif chemokine ligand 8, CYP24A1 - cytochrome P450 family 24 subfamily A member 1, CYP27B1 - cytochrome P450 family 27 subfamily B member 1, CYR61 - cysteine-rich angiogenic inducer 61, DES – desmin, FAP – fibroblast activation protein, FGF2 – fibroblast growth factor 2, FGF7 – fibroblast growth factor 7, FN1 - fibronectin 1, HAS2 - Hyaluronan synthase 2, HGF – hepatocyte growth factor, IDO1 – idoleamine 1, IGF2 - insulin like growth factor 2, IL1B – interleukin 1B, IL6 – interleukin 6, LAMA1 - laminin subunit alpha 1, LOX – lysyl oxidas, MMP11 – metalloproteinase 11, MMP1 – metalloproteinase 1, MMP2 – metalloproteinase 2, MMP3 - metalloproteinase 3, MMP9 – metalloproteinase 9, NFKB1 - nuclear factor kappa b subunit 1, PDGFA - platelet derived growth factor subunit A, PDGFB - platelet derived growth factor subunit B, PDPN – podoplanin, PECAM1 - platelet endothelial cell adhesion molecule 1, PLAU - urokinase-type plasminogen activator, PTEN - phosphatase and tensin homolog deleted on chromosome ten, PTGES2 - prostaglandin E synthase 2, PTGS2 - prostaglandin-endoperoxide synthase 2, RARA - retinoic acid receptor α, RXRA - retinoid X receptor α, S100A4 - S100 calcium binding protein A4, SHH - sonic hedgehog homolog, SPARC - secreted protein acidic and cysteine rich, SPP1 – secreted phosphoprotein, TGFB1 – transforming growth factor β1, TGFBR2 - transforming growth factor β receptor II, THBS1 - thrombospondin 1, THEM4 - thioesterase superfamily member 4, THY1 - Thy-1 cell surface antigen, TIMP1 – tissue metalloproteinase inhibitor 1, TNC – tenascin C, TP53 – tumor protein p53, TSLP - thymic stromal lymphopoietin, VDR – vitamin D receptor, VEGFA – vascular endothelial growth factor A.

Table S 4. **CAFs statuses in tumor tissues from patients with different clinical characteristics**.

|  | **Menopausal status** | | **Vitamin D_3_ level** | | **Metastases** | |
| --- | --- | --- | --- | --- | --- | --- |
|  | **pre-menopausal** | **post-menopausal** | **deficient** | **normal** | **present** | **absent** |
| **No. of patients** | 23 | 51 | 52 | 22 | 27 | 47 |
| **Range of CAFs activation levels** | 1-5 | 1-5 | 1-5 | 1-5 | 1-5 | 1-5 |
| **Mean activation level ± SD** | 2.61 ± 1.20 | 2.24 ± 1.36 | 2.50 ± 1.41 | 2.00 ± 1.02 | 2.44 ± 1.28 | 2.30 ± 1.35 |
| **Range of CAFs density** | 1-5 | 1-5 | 1-5 | 1-5 | 1-5 | 1-5 |
| **Mean of density ± SD** | 2.35 ± 1.53 | 1.96 ± 1.31 | 2.17 ± 1.45 | 1.86 ± 1.21 | 1.74 ± 1.32 | 2.28 ± 1.39 |
| **Range of CAFs infiltration SCORE** | 1-5 | 1-5 | 1-5 | 1-5 | 1-5 | 1-5 |
| **Mean of SCORE ± SD** | 2.35 ± 1.27 | 2.02 ± 1.19 | 2.21 ± 1.26 | 1.91 ± 1.11 | 2.23 ± 1.27 | 1.93 ± 1.11 |

Data presented as mean ± SD. Statistical analysis were carried out using U Mann-Whitney test. * p <0.05 as compared within group (menopausal status, vitamin D_3_ level or metastases).


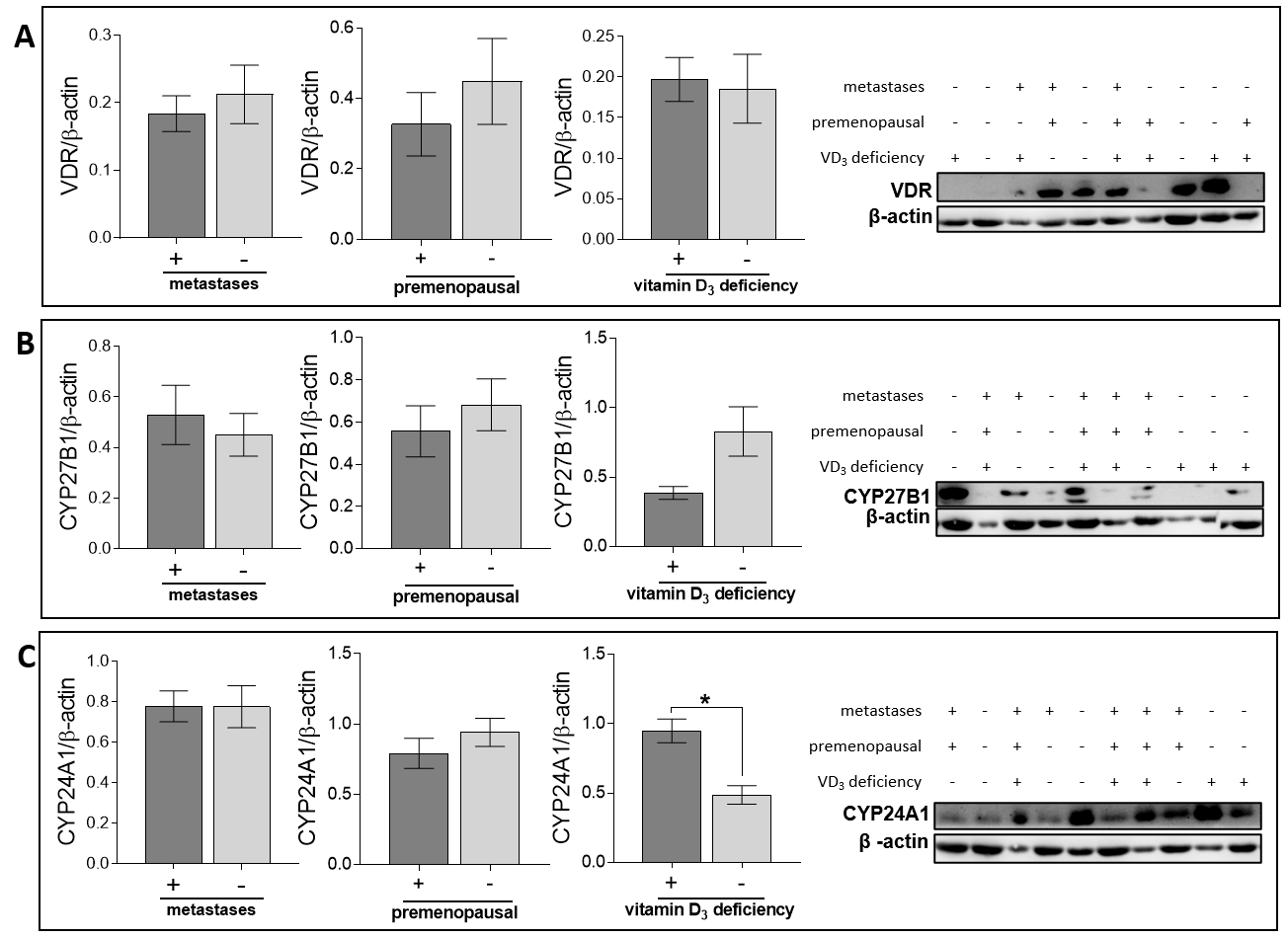


Figure S 1. **VDR, CYP27B1, CYP24A1 levels in tumor tissues from patients with different clinical characteristics**. A – Comparison of VDR (Vitamin D Receptor) levels in tumors from patients with different clinical characteristics. B - Comparison of CYP27B1 (cytochrome P450 family 27 subfamily B member 1) levels in tumors from patients with different clinical characteristics. C - Comparison of CYP24A1 (cytochrome P450 family 24 subfamily A member 1) levels in tumors from patients with different clinical characteristics. Representative cropped blots on tumors from 10 patients are shown next to the graphs. Molecular weight of analyzed proteins: VDR – 48 kDa, CYP27B1 – 58 kDa, CYP24A1 – 59 kDa. Full-length blots are presented in Figure S3 in the Supplementary Material. Patients were classified into groups according to plasma 25(OH)D_3_ levels (VD_3_, <30 ng/mL – deficiency (n=57), >30 ng/mL – normal (n=22)), plasma FSH levels (<25.8 mIU/mL – premenopausal (n=28), >25.8 mIU/mL – postmenopausal (n=52)) and regional or distant metastasis presence (if any – present (n=23), otherwise absent (n=57)). Densitometric analysis was performed using ImageJ software. The results were normalized to β-actin levels. Data are presented as the mean ± SD. Statistical analysis was carried out using Student’s t test or the Mann‒Whitney U test. * p ≤ 0.05.


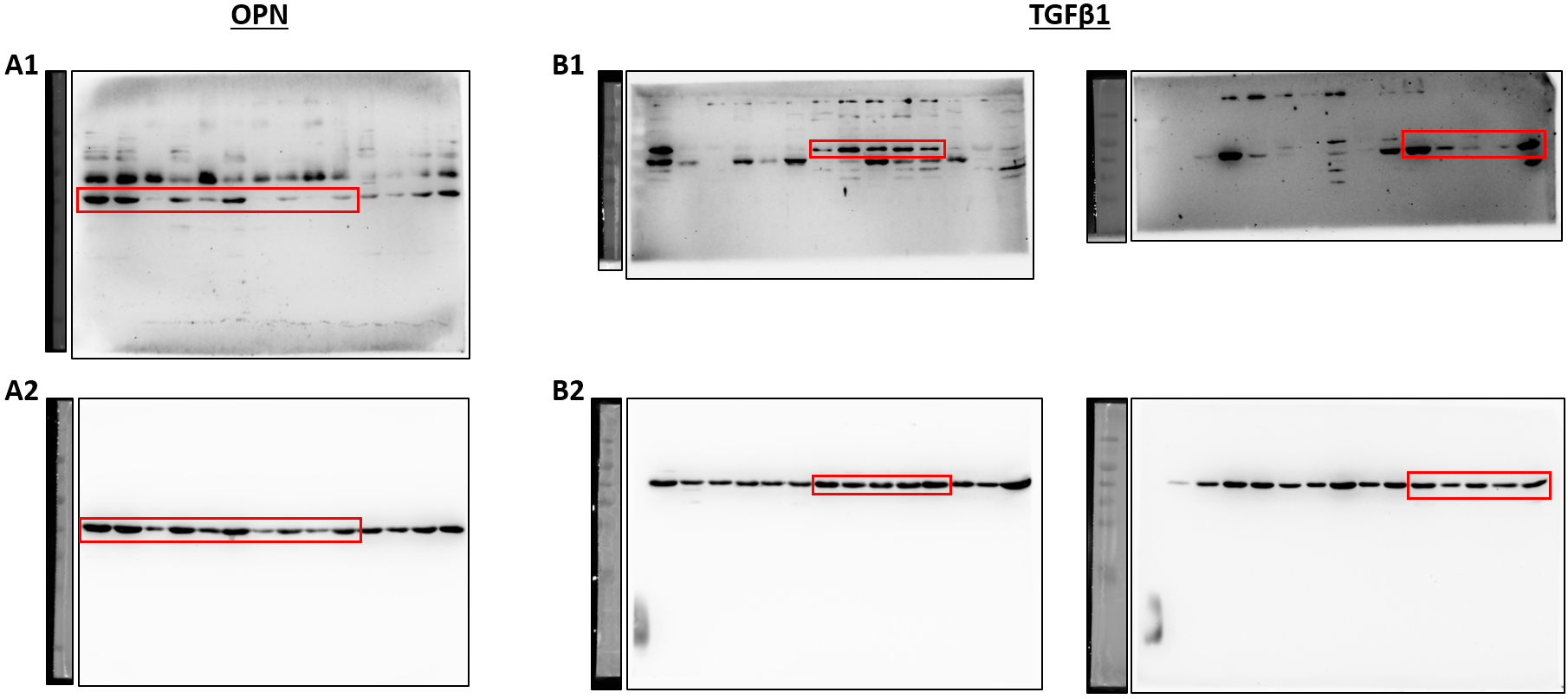


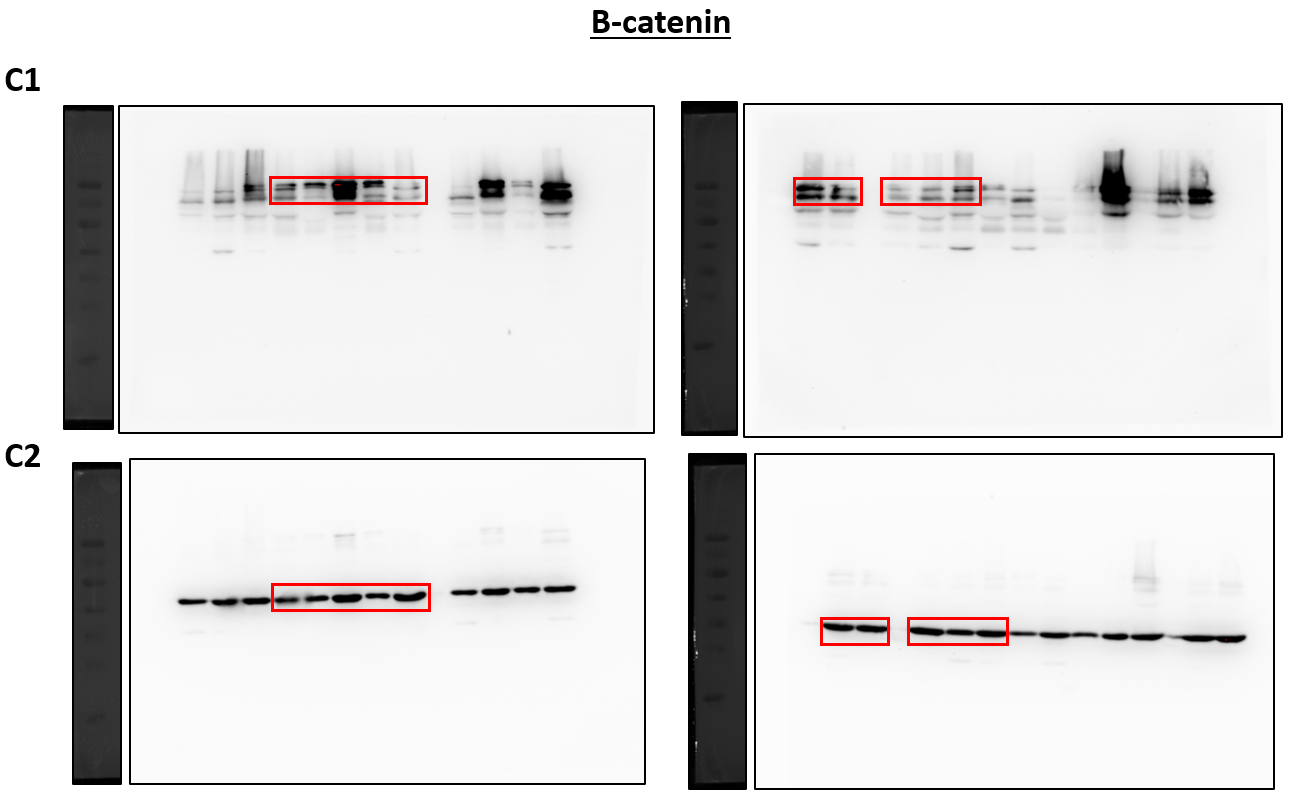


Figure S 2. **Uncropped blot images corresponding to cropped blots presented in Figure 1 in the manuscript:** OPN, TGFβ and β-catenin levels in tumor tissues from patients with different clinical characteristics. A – OPN (osteopontin), B – TGFβ1 (transforming growth factor β1), blot covered during acquisition, C – β-catenin. A1, B1, C1 – Chemiluminescence photography of analyzed protein and fragment of colorimetric photography of the molecular marker. A2, B2, C2 – Chemiluminescence photography of β-actin and fragment of colorimetric photography of the molecular marker.


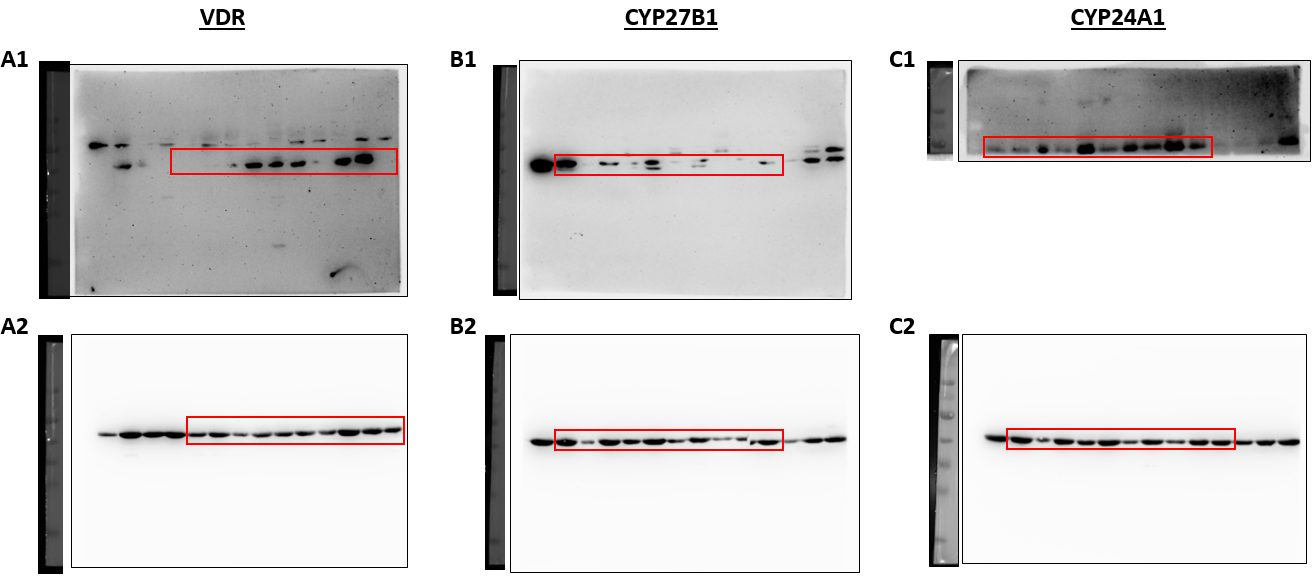


Figure S 3**. Uncropped blot images corresponding to cropped blots presented in Figure S 1 in the Supplementary Materials:** VDR, CYP27B1, CYP24A1 levels in tumor tissues from patients with different clinical characteristics*.* A – VDR (Vitamin D Receptor), B – CYP27B1 (cytochrome P450 family 27 subfamily B member 1), C – CYP24A1 (cytochrome P450 family 24 subfamily A member 1), blot covered during acquisition. A1, B1, C1 – Chemiluminescence photography of analyzed protein and fragment of colorimetric photography of the molecular marker. A2, B2, C2 – Chemiluminescence photography of β-actin and fragment of colorimetric photography of the molecular marker.


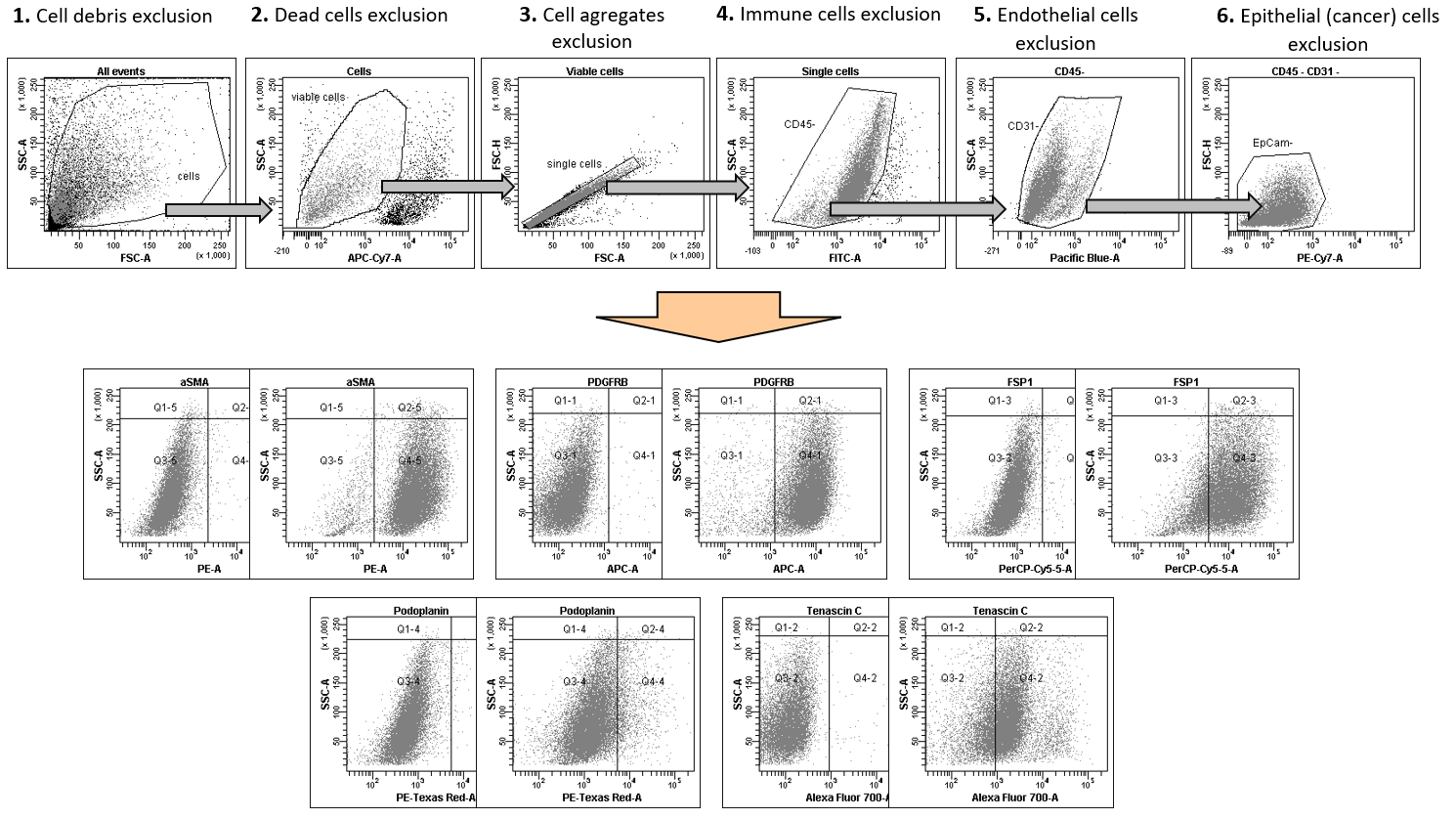


Figure S 4. **Gating strategy for CAFs phenotype assessment using flow cytometry**.


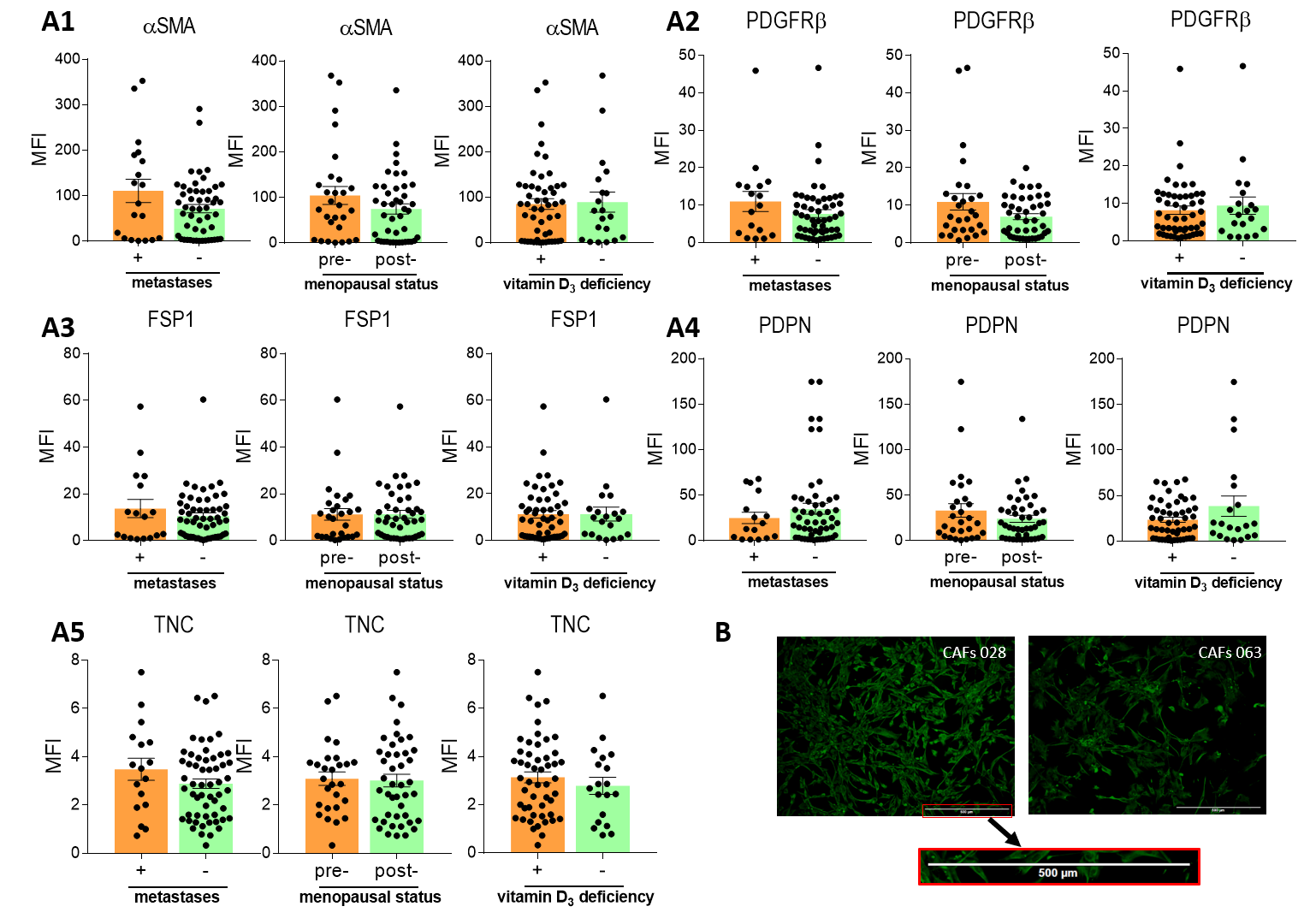


Figure S 5. **Characterization of CAFs phenotype**. A – α-smooth muscle actin (αSMA), podoplanin (PDPN), platelet-derived growth factor receptor β (PDGFRβ), tenascin C (TNC) and fibroblasts specific protein 1 (FSP1) levels in CAFs derived from tumors of patients with different clinical characteristics. B - Vimentin staining in CAFs culture. Representative photos. A - CAFs were analyzed using flow cytometry right after isolation. EpCAM- CD31- CD45- cells were analyzed. Patients were classified into groups according to: plasma 25(OH)D3 levels (<30ng/mL – deficient (n=51), >30ng/mL – normal (n=20)), plasma FSH levels (<25,8 mIU/mL – premenopausal (n=28), >25,8 mIU/mL – postmenopausal (n=43)) and regional or distant metastases presence (if any – present (n=19), otherwise absent (n=52)). Proteins’ levels were presented as median fluorescence normalized to untreated control. Data are presented as mean ± SD. Statistical analysis was carried out using Student’s t test or the Mann‒Whitney U test. * p ≤ 0.05.


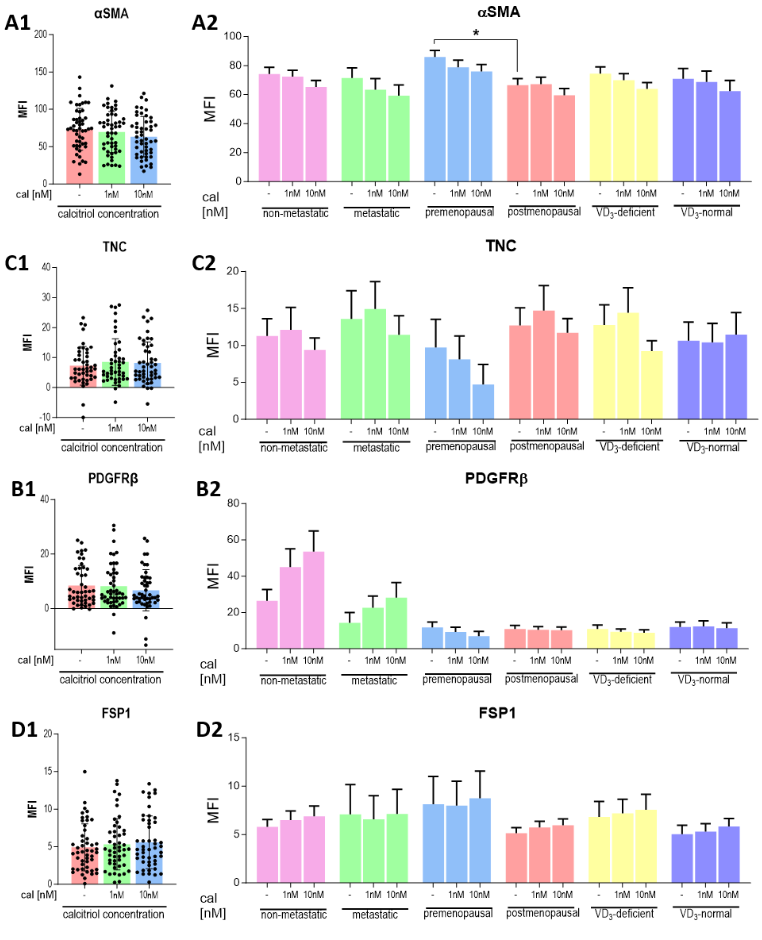


Figure S 6. **Impact of calcitriol on the phenotype of CAFs derived from tumors of patients with different clinical characteristics**. A1-F1 – Data are presented for CAFs from all patients. A2-D2 – Data presented for CAFs from tumors of patients with different clinical characteristics. Levels of following proteins were assessed: A - αSMA (α-smooth muscle actin), B - TNC (tenascin C), C - PDGFRβ (platelet-derived growth factor receptor β) and D - FSP1 (fibroblasts specific protein 1). CAFs were treated with calcitriol (cal; 1 nM or 10 nM) for 72 hours. Patients were classified into groups according to plasma 25(OH)D3 levels (VD3, <30 ng/mL – deficient (n=34), >30 ng/mL – normal (n=17)), plasma FSH levels (<25.8 mIU/mL – premenopausal (n=18), >25.8 mIU/mL – postmenopausal (n=33)) and regional or distant metastasis presence (if any – metastatic (n=16), otherwise non-metastatic (n=35)). Protein levels are presented as median fluorescence (MFI) normalized to the untreated control. Data are presented as mean ± SEM. Statistical analysis was carried out using Student’s t test or the Mann‒Whitney U test for single comparisons and one-way ANOVA or the Kruskal‒Wallis test for multiple comparisons. * p ≤ 0.05.


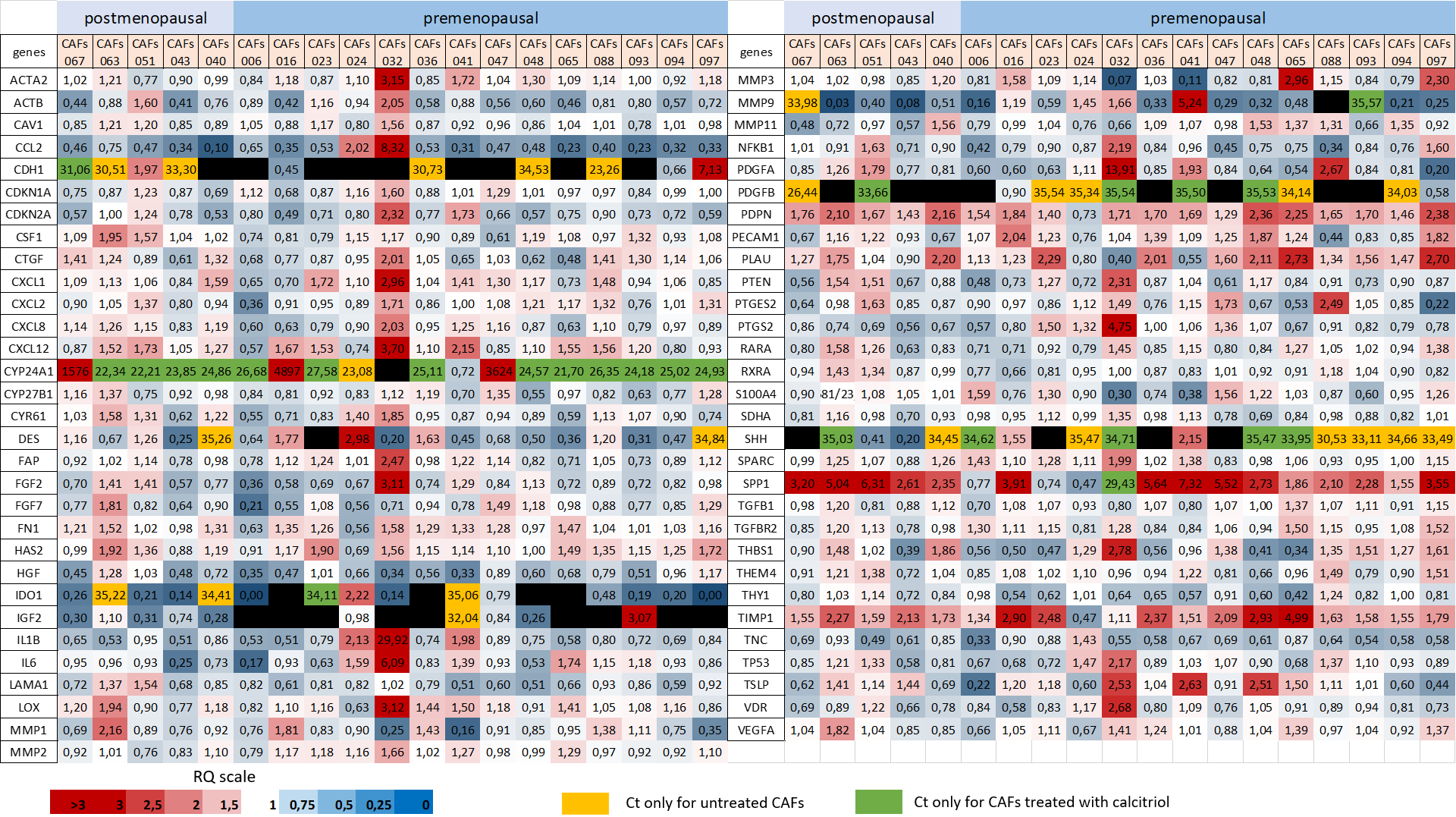


Figure S 7. **The expression matrix of 61 genes from 19 selected CAFs cultures**: 14 CAFs were derived from tumors of premenopausal patients and 5 from tumors of postmenopausal patients. CAFs were treated with 10nM calcitriol for 72 hours. mRNA relative quantity (RQ) was calculated according to ΔΔCt method in relation to endogenous controls (GADPH and RPLP0) and calibrator (untreated control) using QuantStudio™ Real-Time PCR Software and ExpressionSuite Software. In case of CAFs where RQ was not calculated because cycle threshold (Ct) data was obtained only for untreated (yellow) or calcitriol-treated (green) cells, Ct values are presented. Black color represents samples for which any Ct value was not obtained during analysis.


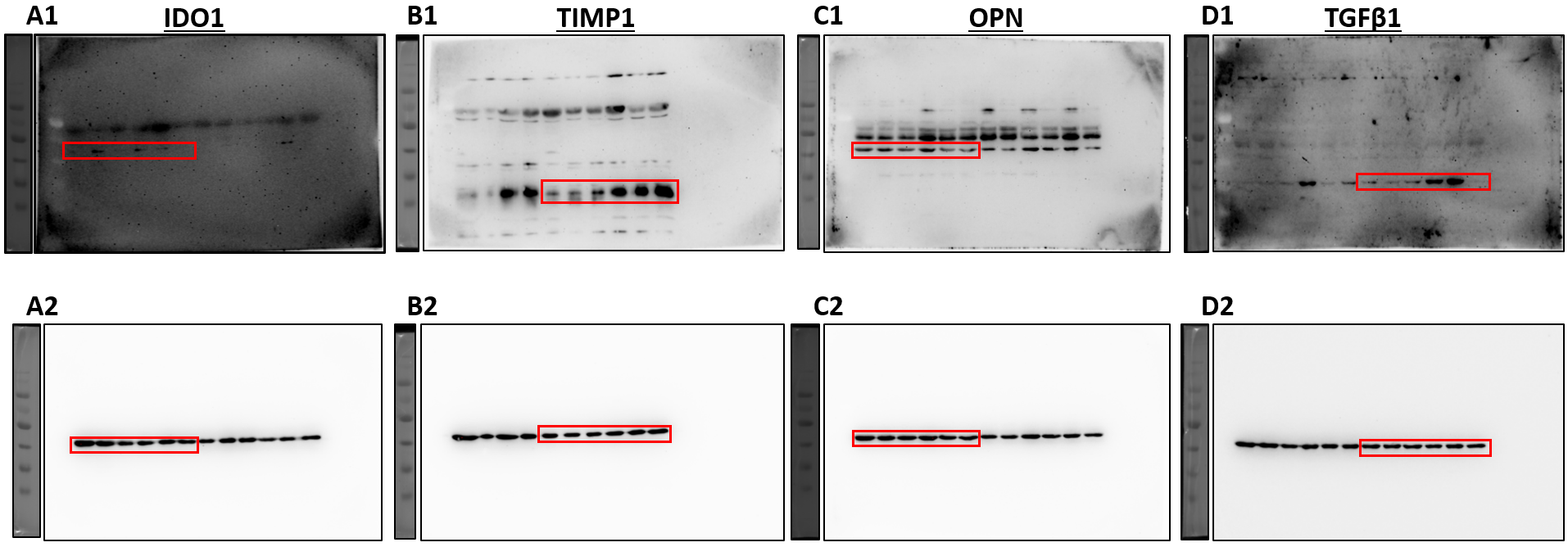


Figure S 8. **Uncropped blot images corresponding to cropped blots presented in Figure 7 in the manuscript:** Selected protein levels in calcitriol-treated CAFs derived from tumors of patients with different clinical characteristics.. A – IDO1 (idoleamine 1), B – TIMP1 (tissue metalloproteinase inhibitor 1), C – OPN (osteopontin), D - TGFβ1 (transforming growth factor β1). A1, B1, C1 – Chemiluminescence photography of analyzed protein and fragment of colorimetric photography of the molecular marker. A2, B2, C2 – Chemiluminescence photography of β-actin and fragment of colorimetric photography of the molecular marker.


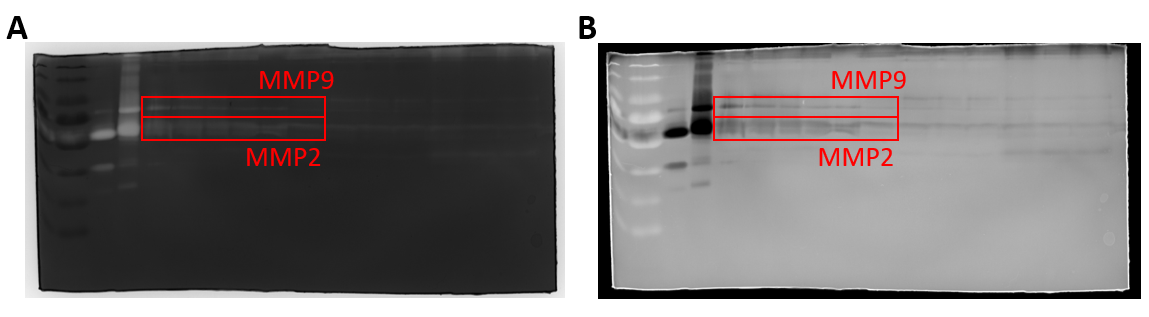


Figure S 9. **Uncropped gel images corresponding to cropped gels presented in Figure 8 in the manuscript:** Gelatinase activity in calcitriol-treated CAFs derived from tumors of patients with different clinical characteristics. A – Original photography, B – Negative of original photography. MMP2 – metalloproteinase 2, MMP9 – metalloproteinase 9.


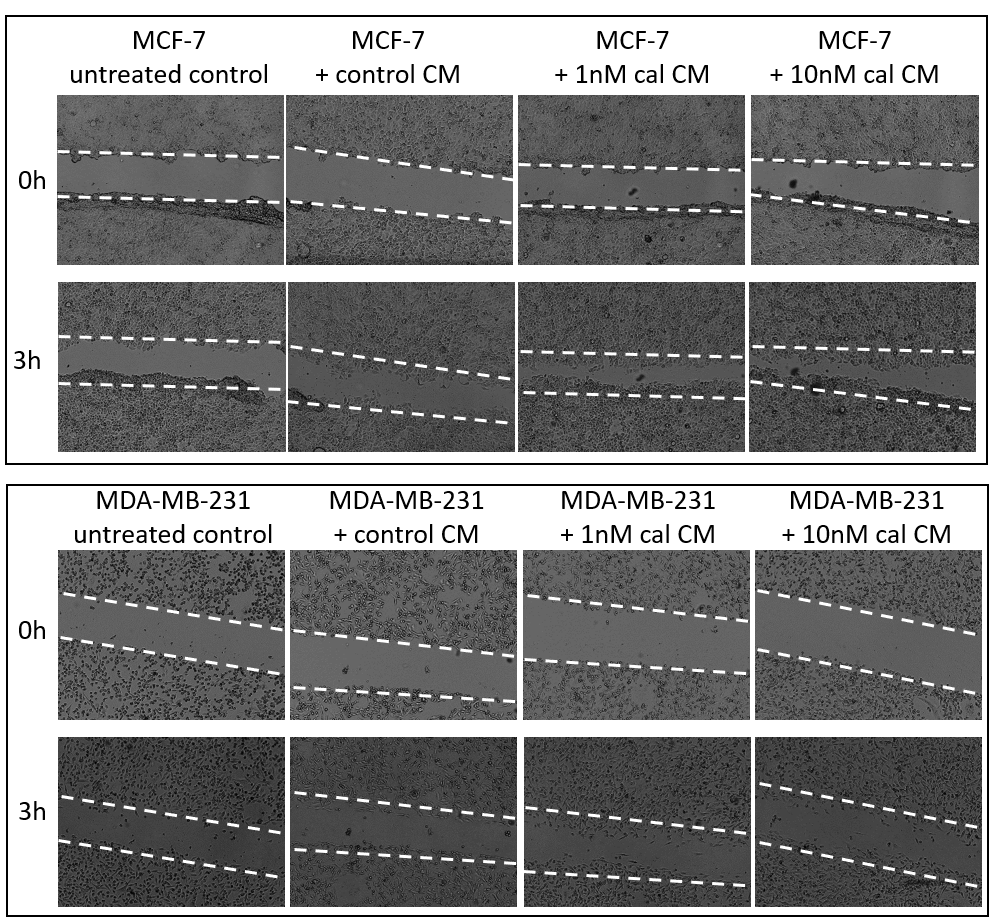


Figure S 10**. Representative photos of the migration of breast cancer cells incubated with conditioned media (CM) from calcitriol-treated CAFs**. CAFs were stimulated with calcitriol (cal) for 72h prior to CM generation for 24h. CM was applied on cancer cell right after wound generation. Cells were photographed in time 0 and 3h after CM application using Stream Start 1.6.1 software.


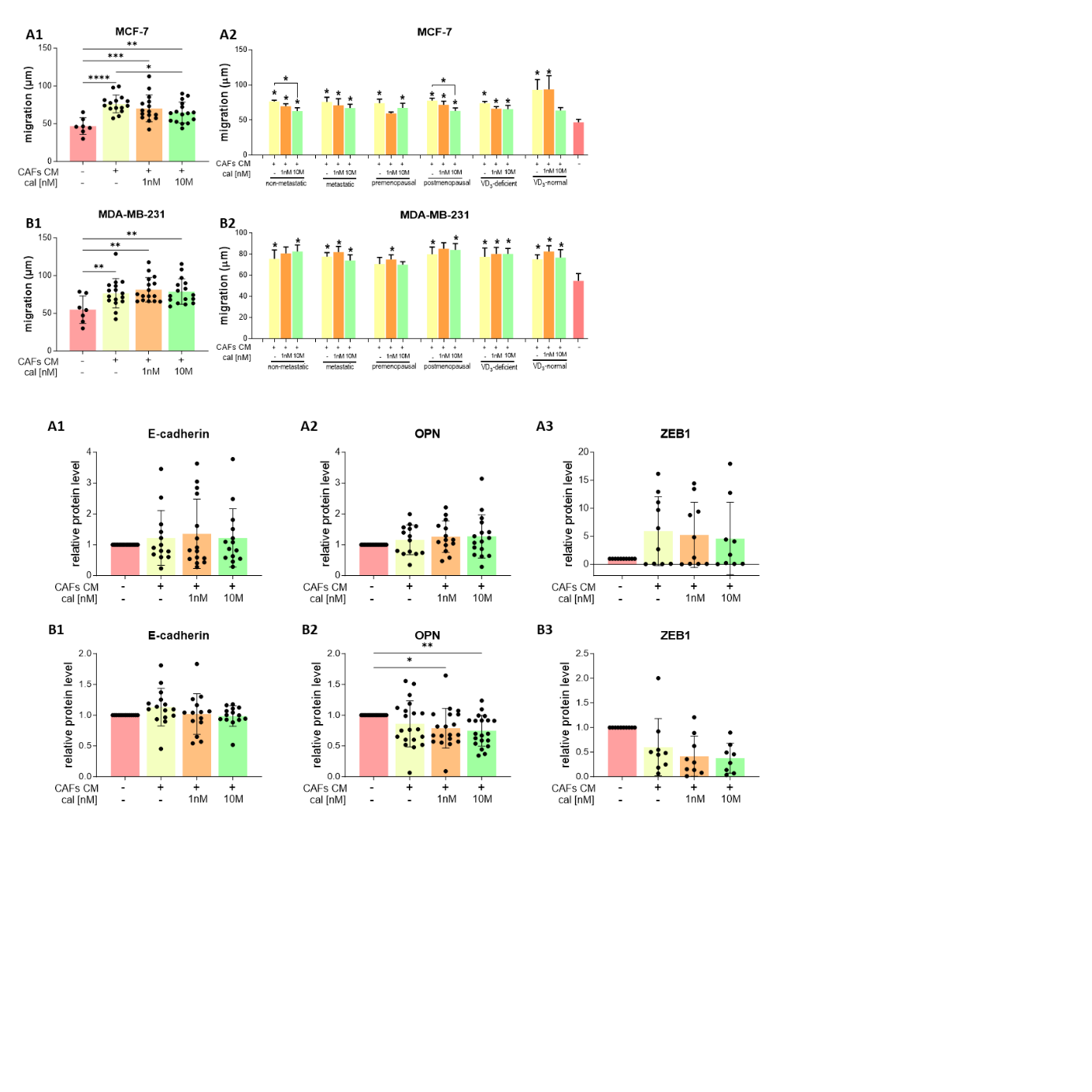


Figure S 11. **Impact of CAFs on the levels of selected proteins in breast cancer cells**. Breast cancer cells: A - MCF-7 and B - MDA-MB-231 were incubated with conditioned media (CM) from calcitriol-treated CAFs derived from tumor of patients with different clinical characteristics. Levels of following proteins were assessed: A1, B1 – E-cadherin levels, A2, B2 – OPN (osteopontin) levels, A3, B3 – ZEB1 (zinc finger E-box binding homeobox 1) levels, Molecular weight of analyzed proteins: E-cadherin – 120 kDa (MCF-7) or 40 kDa (intracellular domain, MDA-MB-231), OPN – 40 kDa, ZEB1 – 130 kDa. Full-length blots are presented in Figure S12 and Figure S13 in the Supplementary Materials. CAFs were stimulated with calcitriol (cal) for 72h prior to CM generation for 24h. CM was applied on cancer cell for 72h. Densitometric analysis was performed using ImageJ software. Results were normalized to β-actin levels and untreated cancer cells. Data are presented as mean ± SEM. Statistical analysis was carried out using one-way ANOVA or the Kruskal‒Wallis test. * p ≤ 0.05.


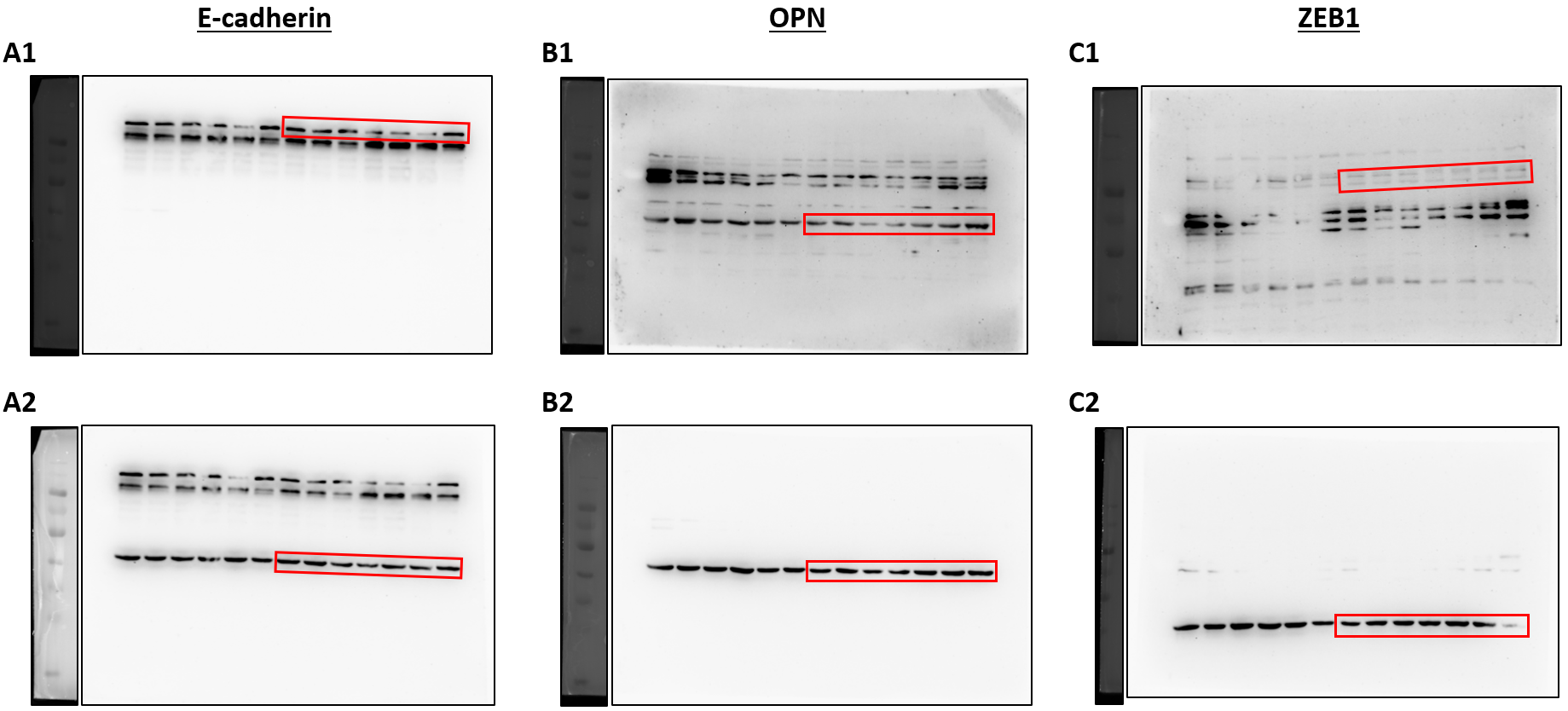


Figure S 12. **Uncropped blot images corresponding to the cropped blots presented in Figure 10 in the manuscript:** CAF impact on the levels of selected proteins in breast cancer cells: MCF-7. A – E-cadherin, B – OPN (osteopontin), C – ZEB1 (zinc finger E-box binding homeobox 1). A1, B1, C1 – Chemiluminescence photography of analyzed protein and fragment of colorimetric photography of the molecular marker. A2, B2, C2 – Chemiluminescence photography of β-actin and fragment of colorimetric photography of the molecular marker.


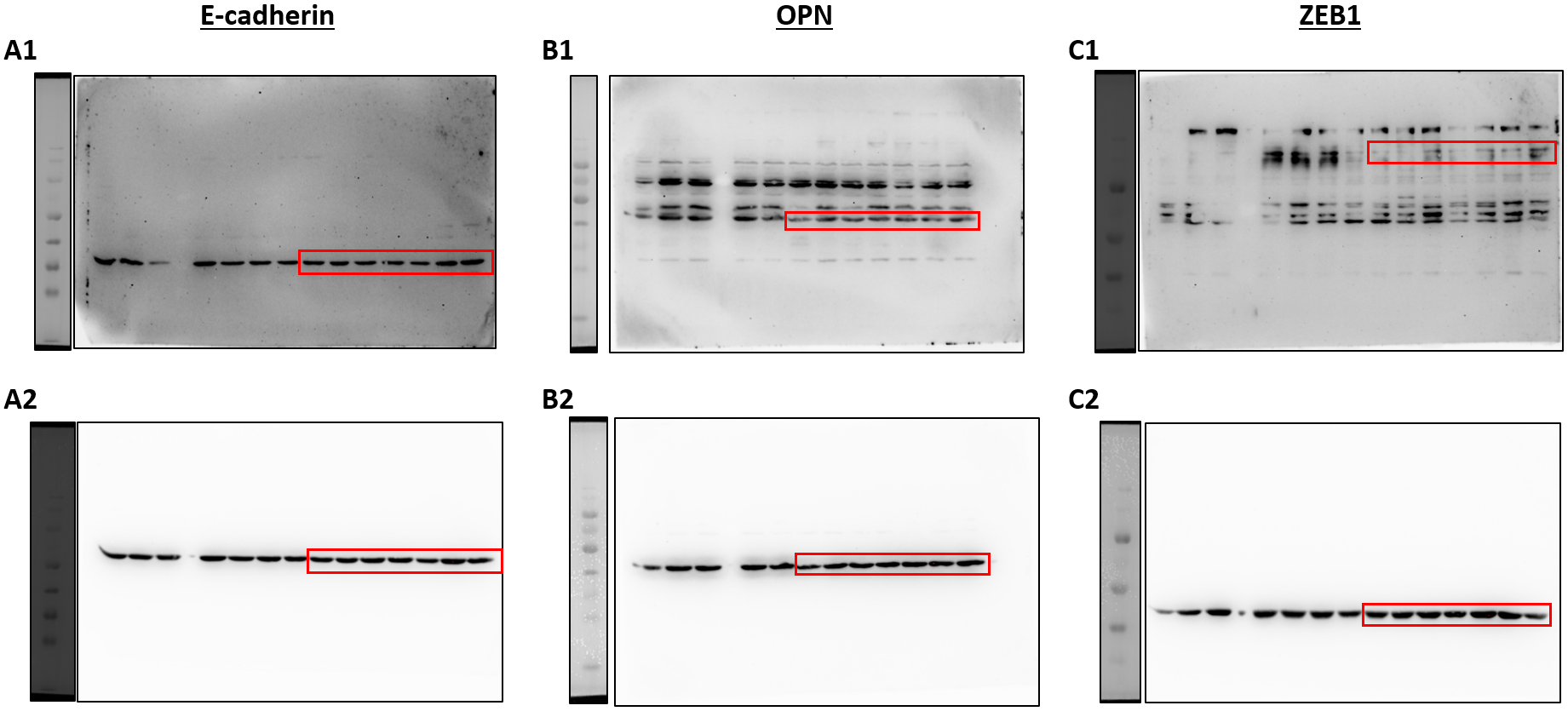


Figure S 13. **Uncropped blot images corresponding to the cropped blots presented in Figure 10 in the manuscript:** CAF impact on the levels of selected proteins in breast cancer cells: MDA-MB-231. A – E-cadherin, B – OPN (osteopontin), C – ZEB1 (zinc finger E-box binding homeobox 1). A1, B1, C1 – Chemiluminescence photography of analyzed protein and fragment of colorimetric photography of the molecular marker. A2, B2, C2 – Chemiluminescence photography of β-actin and fragment of colorimetric photography of the molecular marker
